# Formal Syntax and Deep History

**DOI:** 10.3389/fpsyg.2020.488871

**Published:** 2020-12-18

**Authors:** Andrea Ceolin, Cristina Guardiano, Monica Alexandrina Irimia, Giuseppe Longobardi

**Affiliations:** ^1^Department of Linguistics, University of Pennsylvania, Philadelphia, PA, United States; ^2^Dipartimento di Comunicazione ed Economia, Università di Modena e Reggio Emilia, Reggio Emilia, Italy; ^3^Department of Language and Linguistic Science, University of York, York, United Kingdom

**Keywords:** phylogenetics, formal syntax, parameters, language reconstruction, biolinguistics

## Abstract

We show that, contrary to long-standing assumptions, syntactic traits, modeled here within the generative biolinguistic framework, provide insights into deep-time language history. To support this claim, we have encoded the diversity of nominal structures using 94 universally definable binary parameters, set in 69 languages spanning across up to 13 traditionally irreducible Eurasian families. We found a phylogenetic signal that distinguishes all such families and matches the family-internal tree topologies that are safely established through classical etymological methods and datasets. We have retrieved “near-perfect” phylogenies, which are essentially immune to homoplastic disruption and only moderately influenced by horizontal convergence, two factors that instead severely affect more externalized linguistic features, like sound inventories. This result allows us to draw some preliminary inferences about plausible/implausible cross-family classifications; it also provides a new source of evidence for testing the representation of diversity in syntactic theories.

## Introduction

### The Conceptual Roots of Parametric Comparison

A theory of human language aiming to be part of cognitive science (see Everaert et al., 2015) should try to argue that the structural representations it proposes are: (i) learnable under realistic acquisition conditions; (ii) historically transmitted under the conditions normally expected for the propagation of culturally selected knowledge. The classical theory of generative grammars set itself (i), i.e. the *ontogenetics* of grammars, as its main standard (explanatory adequacy, Chomsky, 1964). We believe that (ii), the *phylogenetics* of grammars, may also provide crucial evidence for the problem of realistic grammatical representations; thus, we test a theory of syntactic diversity inspired by minimalist biolinguistics precisely against the standard in (ii).

### Our Goals

We explore the relationship between the historical signal of different levels of linguistic analysis (referred to as *Humboldt’s* problem by Longobardi and Guardiano, 2009, and as the problem of the *fabric* of human history by Gray et al., 2010; also see Greenhill et al., 2017). For this purpose, we especially try to assess the historical tree-likeness (the problem of the *shape*, in Gray et al.’s 2010 terms) of syntax. In pursuing these goals, we combine some methods of the quantitative revolution in phylogenetic linguistics^1^ with the deductive approach to syntactic diversity that has emerged since Chomsky (1981), and we ask if formal syntactic differences can serve as effective characters for taxonomic purposes, contrary to a long line of skepticism.

### Syntax, Cognitive Science, and Historical Taxonomy

Over the past decades, increased attention has been paid to deep-time investigations of human history.^2^ A central role in this trend has been played by developments in biology, prompted by the use of genetic evidence for reconstructing the diversification of populations.^3^ In the meantime, the rise of cognitive science has produced important breakthroughs in the understanding of human mind as a system of symbolic computations, instantiated e.g., by rules of natural language syntax, most notably in the so-called formal biolinguistic framework.^4^ Against this background, a broad methodological question is: can modern cognitive science side with biological anthropology in contributing to a science of long-range history?

As a matter of fact, the study of language pioneered deep historical investigation: linguistic taxonomies and the discovery of remote proto-languages have crucially contributed to pushing back the time limits of human history and prehistory. However, the levels of linguistic analysis that have best substantiated recent cognitive and computational theories have not yet played a part in this enterprise, and the practitioners of formal grammar and phylogenetic linguistics have formed nearly disjoint communities of scholars. In particular, syntax has never been seriously used for reconstructing phylogenies and proto-languages. Morpurgo-Davies (1992/2014) stresses how the earliest researchers^5^ already rejected syntax as a tool for language phylogeny on the grounds that it would entail the presence of similar features in languages that can be easily proved to be unrelated, i.e., that it would be subject to pervasive homoplasy.^6^ Since the late 18th century, this assumption appears not to have changed, even after Kayne (1975) laid the basis of modern comparative syntax. Consider, for instance the following statement:

(1) “In fact it is quite possible – even likely – that English grammars might be more similar to grammars with which there is less historical connection. From this perspective, looking at the parameters in the current linguistic literature, English grammars may be more similar to Italian than to German, and French grammars may be more similar to German than to Spanish. *There is no reason to believe that structural similarity should be even an approximate function of historical relatedness*…”
  (Anderson and Lightfoot, 2002, pp. 8–9: our italic)

### The Historical Signal of Syntax

Positions along these lines are widely held in the field (cf. Newmeyer, 2005; Anderson, 2012, a.o.).^7^

Interestingly, at a small scale it is commonly accepted that syntactic variability aggregates across individuals in time and space.^8^ For instance, an important facet of the logical problem of language acquisition (Hornstein and Lightfoot, 1981; Lightfoot, 1982, a.o.) makes crucial reference to this kind of similarity among I-languages (how do the children of *a community* converge on the *same* target grammar in certain subtle details, in spite of individual and idiosyncratic primary data?).

It is at a larger scale (e.g., of Romance or Indo-European) that this simple assumption becomes progressively controversial, neglected or altogether rejected, for non-obvious reasons. Normally, culturally transmitted phenomena leave a longer-term historical trace (e.g., some notion of “common Romance vocabulary”). Therefore, that even syntax does so should be the null hypothesis.

It is true that individual syntactic changes may be “catastrophic” and unpredictable: this discovery (Lightfoot, 1979, 1997, 2002, a.o.)^9^ has been very instrumental in overcoming the epistemological pitfalls of classical linguistic historicism and reducing inquiry to its appropriate “molecular” units: individual parameters. Yet, if several syntactic parameters are considered at the same time, a historical signal might well emerge. Notice that if such a signal were completely irretrievable, then someone could even argue that generative syntax is inadequate as a model of language transmission (i.e., acquisition across generations), hence as a realistic cognitive model *tout court*.^10^

### Syntactic Data and Taxonomic Problems

Two general problems of linguistic taxonomic methods (cf. Guardiano et al., 2020) are especially relevant for our purposes:

(2) a. The *globality* problem
b. The *ultralocality* problem

(2)a refers to the fact that comparative procedures may aspire to long-range or, ideally, global coverage; thus, they should rely on universally definable taxonomic characters, that can apply to any set of languages. (2)b is the converse issue: even if some type of characters does not saturate at the macro-comparative level, it could still fail in resolution when applied to discriminate close dialects, or just fail to correlate altogether with the reduction of their differences in other linguistic aspects.

Even if promising advances in cross-family comparison have recently been made (Jäger, 2015), procedures based on vocabulary data and lexical arbitrariness are generally not appropriate for (2)a, because they mainly rely on family-internal etymologies.^11^ Therefore, the development of a non-lexical method is a theoretical *eldorado* in the pursuit of deep language history (Nichols, 1992). Parameters in the theory of generative grammars should lend themselves well to this goal, as they are grounded in a model of the language faculty explicitly designed in universal terms.

Thus, we focused on: (i) a set of syntactic traits modeled along the lines of Longobardi and Guardiano’s (2009) Parametric Comparison Method (PCM) and including macro-, meso-, and micro-parameters (Biberauer and Roberts, 2017; Roberts, 2019);^12^ (ii) a language sample to test these traits against family-wide taxonomies, but also with respect to cross-family and dialect comparison.

Importantly, we assumed some idealizations about the adopted comparative characters:^13^

(3) a. *Modularity*: they are all purely syntactic traits, drawn from a single module of syntax (the internal structure of nominal phrases);
b. *Deductivity*: they are all coded as abstract primitives of the generative device;
c. *Interdependence*: their known and plausible dependencies are spelt out and built into the parametric structure.

These three properties of our input data are different from those attributed to the structural traits recently used to address similar issues, e.g., in Greenhill et al. (2017). We will explore some consequences of using traits with these three properties for the pursuit of long-range comparison (cf. Section “Input data and phylogenetic results”).

## Materials and Methods

### Parameters and Schemata

In classical Principles-and-Parameters models (Chomsky, 1981) it was assumed that variability in human grammars is reducible to a finite list of binary choices, extensionally present in every speaker’s mind at the initial state of language acquisition. This “preformistic”^14^ view has been criticized recently. In particular, it has been associated with an implausible model of language learnability, as it imposes too heavy a burden on the initial state of the human mind.^15^

Here we ‘presuppose’ a model of variation which does not necessarily rely on lists of parameters, but rather sketches a universal set of simple possible syntactic relations (i.e., *schemata*: Longobardi, 2005, 2014, 2017; Gianollo et al., 2008); whether, in each language, they apply or not to specific categories and features determines a number of binary choices epigenetically rather than preformistically. This minimalist parametric model (Principles and Schemata in Longobardi’s, 2005 terms) has the effect of intensionally defining parameter lists with their familiar properties (including universal definition and ease of value collation for comparative purposes: Roberts, 1998), without attributing such lists extensionally to the common initial state of the language faculty.

Our parameters are formally coded using two symbols, “+” and “−”. Specifically, we adopt the system proposed in Crisma et al. (2020): cognitively, just “+” is viewed as an addition to the initial state of the mind. The “−” state of a parameter is not an entity attributed to the speaker’s mind, though it is used by the PCM as a symbol to code a difference with “+” at that parameter in another language.

We call “manifestation(s)” the empirical evidence that sets a given parameter. Most parameters have a clustering structure, i.e., are associated with a set of co-varying surface manifestations,^16^ with different degrees of saliency. As a consequence of such clustering structure, identifying just one core manifestation (a trigger or *p-expression* in Clark and Roberts’, 1993 sense) per parameter will suffice for the learner (and the linguist) to set the parameter to “+.” If no relevant manifestation for “+” is present in the data, the grammar’s *default* state does not change.

P-expressions are by definition *positive* evidence, i.e., grammatical phrases of a language. In the formulation of the parameters we made sure that the non-default value “+” can be set in all the languages from positive evidence in this sense.

### The Syntactic Dataset

In this article, we used the 94 binary syntactic nominal parameters identified in Crisma et al. (2020) by a set of YES/NO questions which define the manifestations of each of them.^17^ They are set in 69 contemporary Eurasian languages from up to 13 traditionally irreducible families.^18^ Full information about the languages and the parameter states is available in Supplementary Table 1 and Supplementary Figure 1.

The languages were chosen to investigate three different levels of historical depth: the relations of the deepest established families, their internal articulation, and dialect microvariation. To explore the latter, we rely on the sample of Romance^19^ and Greek^20^ dialects included in the dataset.

### Some Numerical Properties of the Syntactic Data

The parameters of our system display an intricate implicational structure (Guardiano and Longobardi, 2017), i.e., many parameter states turn out to be predictable, or completely irrelevant, given the states of other parameters.^21^ In the dataset used in this article, 2925 states out of 94 × 69 (= 6486) are null, perhaps the most impressive instantiation of the insight (sometimes attributed to Meillet, but cf. Toman, 1987) that natural languages are “un système où tout se tient.” The effect of such null states on the number of possible languages has become measurable since Bortolussi et al. (2011), proving to reduce it by several orders of magnitude (cf. Section “Possible Languages” in Supplementary Material).

A related numerical feature of the syntactic dataset is that in a system with two non-null states (“+” and “−”) and a null state (coded as “0” and representing no independent information) the only relevant comparisons for a pair of languages are provided by parameters for which neither language displays a “0”: namely an identity (“+/+” or “−/−”) or a difference (“+/−” or viceversa). The average number of parameters for each language pair that does not display “0” in either language is 39 (in the range of 14 to 66). Thus, the historical signal which can be found in this dataset will be generated by an average of taxonomic characters no higher than 39 (a figure much lower than that of the taxonomic units investigated)^22^ : if a significant signal is indeed found, this will suggest that the selected characters have a high degree of resolution.

From a practical viewpoint, it is also important to stress that, thanks to the structure of the parameter system, in order to fill in the states of the 94 parameters for each language it is only necessary to find positive evidence for the “+” values; this is so because “0” is totally deducible information and “−” is a default state. In our dataset the total amount of “+” is 1386, thus, the mean is 20 “+” per language; the median is also 20. Hence, the amount of parameter values which must be set from positive empirical evidence is only about one quarter of the whole parameter list.^23^

### Taxonomic and Phylogenetic Methods

We have performed a series of experiments using some standard computational tools, although none of them was conceived for − or specifically adjusted to − syntactic, rather than biological or lexical data. Such tools belong to two major types: distance-based and character-based programs.

#### Distance-Based Methods

We used three distance-based tools: heatmaps,^24^ PCoAs,^25^ and UPGMA phylogenetic trees.^26^

Heatmaps can be used to identify clusters in a distance matrix: in the heatmap, each cell (corresponding to a language pair) is assigned a color according to its distance value; then, through a hierarchical clustering algorithm, cells can be arranged on the basis of their color: language pairs which share small distances are arranged along the diagonal of the square matrix.

Principal Coordinate Analyses (PCoAs) represent a distance matrix on a Cartesian plane by plotting the taxa on a bidimensional space, using a linear transformation of the distance matrix.

The distance-based algorithm that is typically used to generate phylogenetic trees from a distance matrix is Neighbor-Joining.^27^ Previous work on syntactic data showed that identifying a root and imposing the same branch length between a root and the leaves (i.e., assuming a molecular clock) through an updated version of Neighbor-Joining (the UPGMA algorithm) improves the classification.^28^ Hence, for our distance-based phylogenetic experiments, we adopted UPGMA (using the package PHYLIP, Felsenstein, 2005).

#### Measuring Syntactic Distances

One of the main challenges about our data is dealing with null characters (“0”). Distance-based methods allow us to do so in a simple way: whenever one of the languages of a pair has a “0” for a certain parameter (cf. Section “Some numerical properties of the syntactic data”), we can just ignore the parameter in calculating the distance of the pair. To deal with this problem, we first normalized a standard distance metric (Hamming, 1950) by dividing, for each pair of languages, the number of differences by the sum of their identities and differences.

Our background parameter theory (cf. Section “Parameters and Schemata”) assumes that, of the two potential states of a parameter, the value “−” instantiates a default state: thus, identities on two “−” should *a priori* be less marked than identities on two “+.” In other words, the former could be less likely than the latter as shared innovations in the phylogenetic history. However, it is difficult to assess the actual weight of the potentially less informative “−/−” correspondences: therefore, we explored the radical idealization of counting as identities only the “+/+” ones. This amounts to using a Jaccard (1901) metric:^29^

(4) Δ Jaccard(A,B) = [N_−+_ + N_+−_]/[N_−+_ + N_+−_ + N_++_]

where N_XY_ indicates the number of positions where the string A has value X and B has Y.

To measure the impact of the idealization, we performed experiments both through a Jaccard distance and a normalized Hamming distance (in which “+/+” and “-/−” are both counted as valid identities) and the results are slightly worse for Hamming^30^ (cf. Section “Phylogenetic Analysis – Hamming Distances” in Supplementary Material); therefore, we decided to simply proceed with the more restrictive Jaccard formula.

The heatmap, the PCoAs and the phylogenetic tree shown in Figure 3 were generated from the Jaccard distance matrix inferred from the parametric characters of Supplementary Figure 1.

#### Character-Based Methods

Character-based methods were specifically devised to reconstruct the sequence of changes in the character states of a dataset.^31^ Character-based phylogenetic methods have mostly been used to calculate linguistic splits and dates.^32^ In particular, Bayesian inference has been recently implemented to evaluate the probability of different evolutionary models: for instance, whether the rate of change is uniform across branches and across characters, or whether it can be modeled according to some mathematical distribution. Evolutionary models are then used to generate phylogenetic trees. We employed the software BEAST 2 (Bouckaert et al., 2019), which is the most up-to-date tool to perform Bayesian phylogenetic analysis.

Finally, we calculated two tree-likeness metrics, Δ-scores and Q-residuals,^33^ from a network generated through the algorithm NeighborNet, from SplitsTree.^34^ These measures estimate the robustness of the vertical signal, and indicate which taxa are weaker due to the possible presence of horizontal convergence or homoplasy.

#### Some Problems With Current Methods

Both methods require some idealization about the data structure, and therefore either methodological choice can be expected to misrepresent some aspect of the information contained in the dataset.

When using distance-based algorithms, reducing all pairs of strings (languages) in the dataset to a distance matrix implies that the exact position of identities and differences between them becomes irretrievable. Moreover, the choice of distance metrics has an impact on how differences are weighted against identities.

Character-based algorithms, on the contrary, are the closest automatic analog to the linguists’ consolidated procedure of reconstructing all ancestral states (e.g., sounds and etymologies) and changes, and of postulating taxa on this basis (Greenhill et al., 2020); however, a straightforward exploitation of their potential for our data is still partly hampered by at least two features of these algorithms.

First, these methods assume character states and their changes to be independent, an assumption which is not true in our case. Therefore, they do not offer any intuitive solution to deal with implied values (“0”), because they were not devised to incorporate interdependence among characters. Coding the state “0” as a third, independent value, would be an arbitrary manipulation of the data, because “0” represents completely predictable information rather than additional information or points of uncertainty.^35^ To mitigate this problem, we coded the implied states (“0”) as missing characters, to allow the algorithm to ignore redundant characters as a source of information.^36^

The second problem is that character-based algorithms are not *a priori* informed about asymmetries in the likelihood of state transitions. Historical phonology clearly shows several cases of this kind: for instance, Honeybone (2016) shows that a change from the voiceless interdental fricative [θ] to the labial fricative [f] is common, but the reverse is virtually unattested outside of contact areas. Other classic examples are [p] > [f], [p] > [h] or [p] > Ø, all recurrent changes in Indo-European and beyond, and [f] *>* [p], [h] > [p] or Ø > [p], all extremely rare. With respect to our parameters, we know that there are, for example, several cases of languages acquiring grammaticalized definiteness and no cases of languages dropping this feature,^37^ something likely to be reduced to principled explanation, based on the combination of general conditions on change like *Inertia* (Keenan, 2002, 2009) and *Resistance* (Guardiano et al., 2016). An efficient character-reconstructing algorithm will have to be eventually endowed with most such information, but this is not yet the case.

We may expect these problems to affect the topology retrieved by such algorithms. As a consequence, on the other side, any positive taxonomic results retrieved by these methods will attest to the robustness of the signal even *in spite of* the present limitations.

## Results

### Distance-Based Experiments

#### Heatmap

**FIGURE 1:**
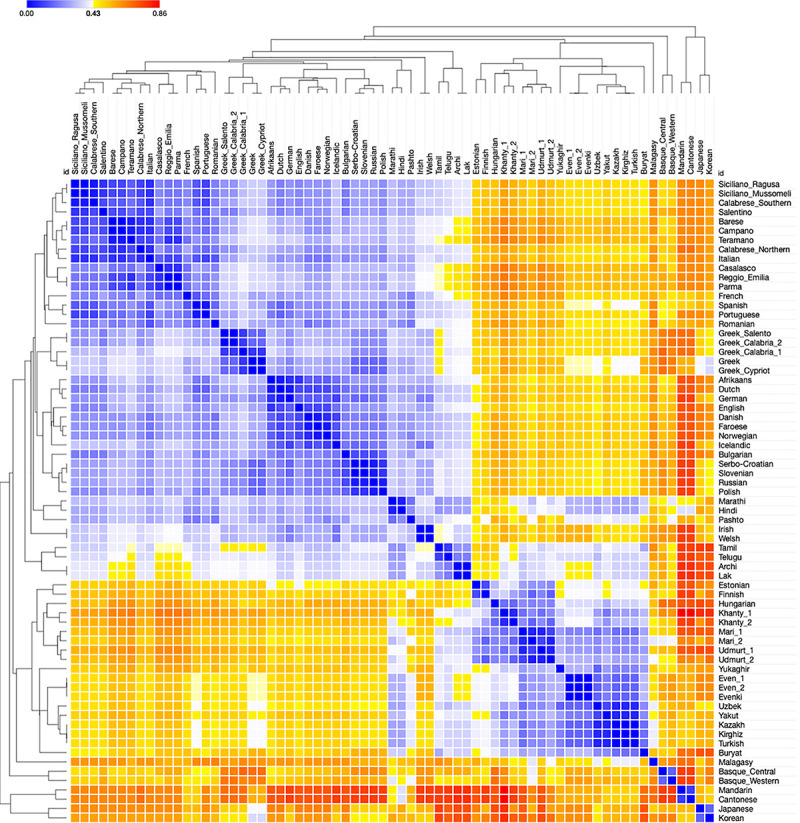
Heatmap of syntactic Jaccard distances between the 69 languages of the sample, calculated on 94 parameters.

The information contained in the syntactic distances was first examined by means of the Heatmap in Figure 1. Colors from white to dark blue signal distances lower than the median (spanning from 0 to 0.429), those from yellow to dark red signal distances higher than the median (spanning from 0.430 to 0.857). The overall distribution of colors in Figure 1 shows that the distances are scattered enough from dark red to dark blue to be potentially informative.

To assess if their distribution has any empirical significance, we considered the maximal aggregations of (white and blue-shaded) cells containing no yellow/red ones which are identified through the clustering option of the program (cf. Section “Distance-based methods”); we compared them to the established genealogical clusters in the sample. In the figure, there are 6 such aggregations which are unambiguous. They correspond to:

(5) a. The Indo-European (henceforth IE) languages.
b. The two Dravidian languages and the two NE-Caucasian ones.
c. Malagasy.
d. The two Basque varieties.
e.  The two Sinitic languages.
f.  Korean and Japanese.

Two further groups of clusters are also identified along the diagonal. They are more ambiguously interpretable, owing to the fact that they display a partial overlap; in principle, they could single out either the groups in (6) or in (7):

(6) a. Uralic.^38^
b. Turkic,^39^ Tungusic,^40^ Buryat (i.e., the languages traditionally attributed to the controversial^41^ Altaic group) and Yukaghir.

(7) a. Balto-Finnic.
b. The rest of Uralic, Tungusic, Buryat, and Yukaghir.

The clustering algorithm suggests that (6) is the more plausible hypothesis, as highlighted in the tree-like structure on the left and top borders. Hence, the distance distribution in the Heatmap only identifies established taxa (families or isolates: (5)a, c,^42^ d, e, (6)a) or supersets of them ((5)b and f; (6)b): thus, no cluster challenges any known historical information, and three of them suggest possible though not yet established supertaxa.

There is also a weaker aggregation of white/pale blue cells next to the sides of the clusters identified along the diagonal. It corresponds to pairs of languages from different families dwelling in the central part of Eurasia (Indo-Iranian, Dravidian, and NE-Caucasian, Altaic, Yukaghir, Uralic except for the three languages now spoken in central and Northern Europe). However, no possible aggregation of white/blue cells displays an average internal distance lower than those of the aggregations identified in (5) and (6) (cf. Supplementary Material).

#### PCoA

**FIGURE 2:**
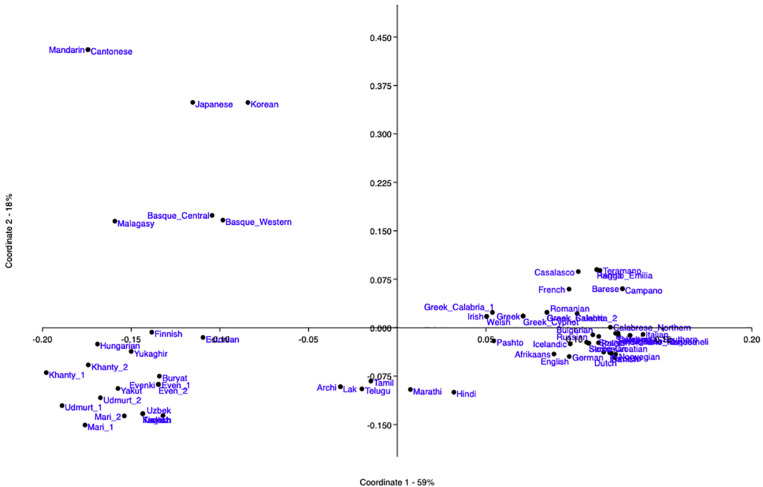
PCoA obtained by the software PAST from the syntactic Jaccard distances between the 69 languages of the sample, calculated on 94 parameters.

The PCoA obtained from the syntactic distances between all the language pairs of the dataset is in Figure 2. The first coordinate, which accounts for 59% of the variance, highlights the split between:

**FIGURE 3:**
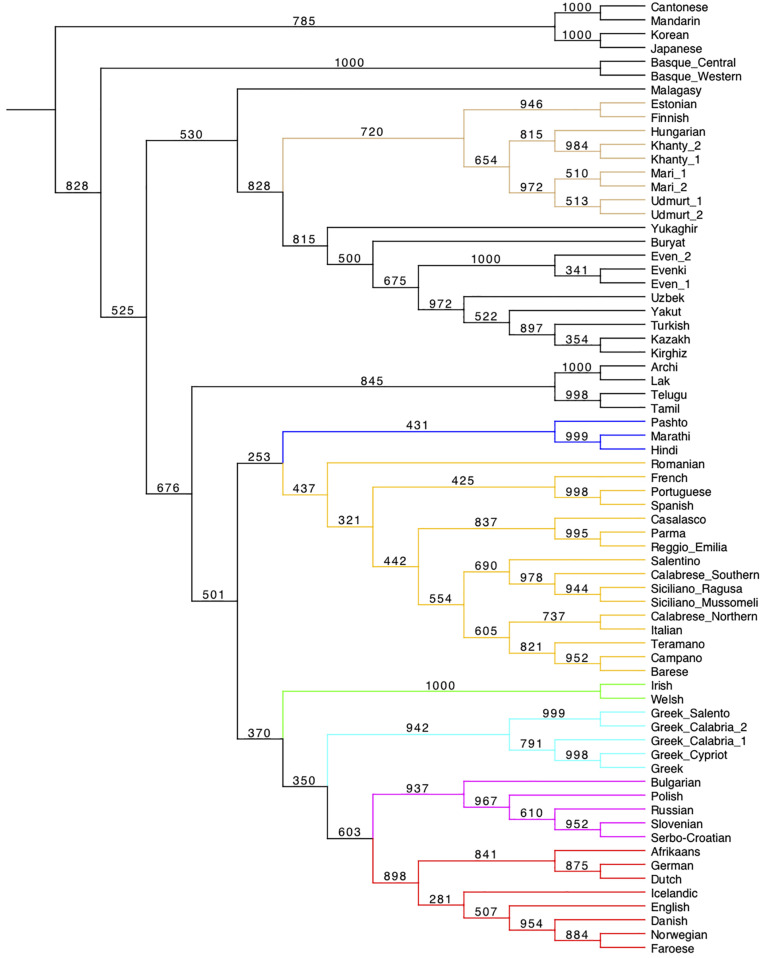
UPGMA tree from syntactic Jaccard distances between the 69 languages of the sample, calculated on 94 parameters. The tree has been produced using Mesquite (Maddison and Maddison, 2007). For information on the bootstrapping procedure adopted, cf. Section “Phylogenetic Analysis – UPGMA” in Supplementary Material.

(8) a. Non-IE languages (left area).
b. IE languages (right area).

In the left half, the further split corresponding to the second coordinate (accounting for 18% of the remaining variance) separates:

(9) a. Upper-left quadrant: the four languages of the Far East, Malagasy (which has known roots in the same area), and the two Basque varieties, in a rather scattered shape.
b. Bottom-left quadrant: all the other languages of the dataset, i.e., a cloud containing Uralic, Altaic, and Yukaghir and another one with Dravidian and NE-Caucasian.

In order to obtain a higher resolution, we generated a sequence of further PCoAs from the various subsets of languages progressively identified by the previous ones (cf. Section “PCoAs” in Supplementary Material), and they continue to distinguish sets and supersets of independently acknowledged taxa.

#### Distance-Based Phylogeny

The tests above have preliminarily suggested that a good deal of syntactic diversity is roughly distributed in agreement with genealogical affiliation. Next, we applied phylogenetic algorithms to our data. Figure 3 displays a (bootstrapped) UPGMA tree. Every cluster identified in the Heatmap also appears in the UPGMA tree.

### Character-Based Experiments

#### Character-Based Phylogeny

**FIGURE 4:**
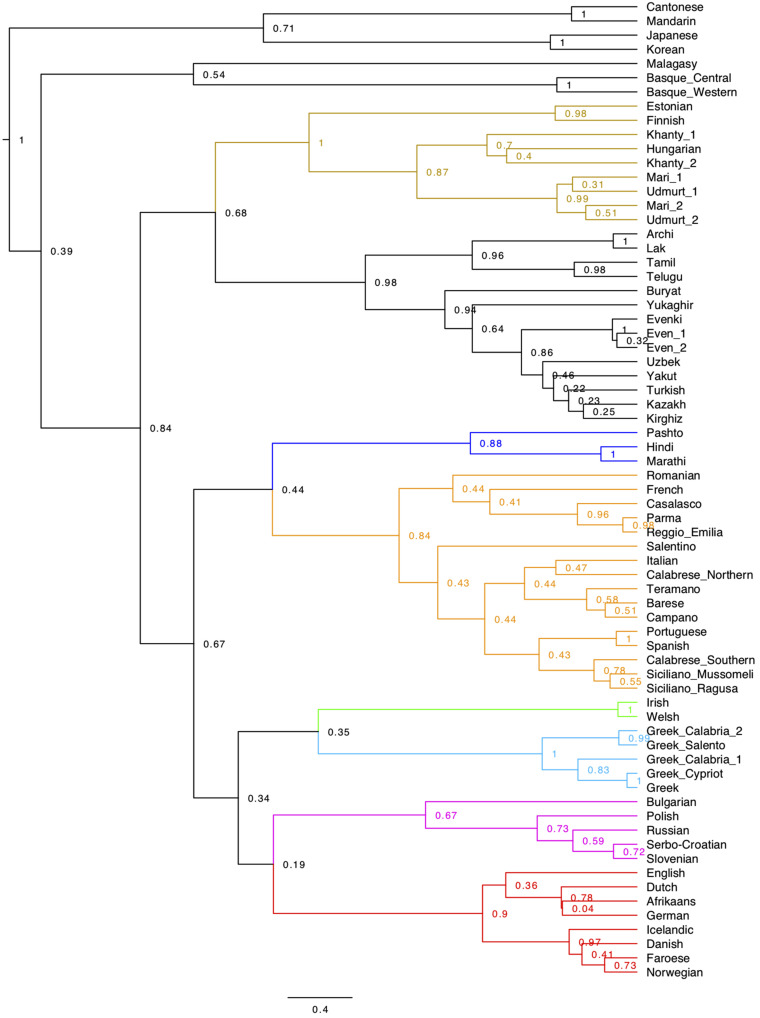
BEAST tree from the 94 syntactic parameters set in the 69 languages of our sample. The best model that we determined is a Gamma Site Model with Substitution Rate = 1, a Mutation Death Model with death *p* = 0.1, a Relaxed Clock (Logarithmic) with clock rate = 1, and a uniform Yule model for the birth rate. The Monte Carlo Markov Chain produced 10,000,000 trees, 25% of which were used for the burn-in and discarded for the purpose of the calculation of the consensus tree. The tree is a consensus tree of 7500 different trees sampled through the 7,500,000 trees (with a sample stored every 1000 generated trees) produced by the Monte Carlo procedure.

The taxonomic results obtained from syntactic distances were finally confirmed by a character-based phylogeny even in spite of the limitations pointed out in Section “Some problems with current methods”. The phylogenetic tree calculated with BEAST is in Figure 4. The best model was determined by comparing different models using the software Tracer (cf. Section “Phylogenetic Analysis – BEAST 2” in Supplementary Material). We noticed that most of the nodes were robust across different replications, and the variation was limited to the lower nodes, but a salient exception was the node grouping together Finnish and Estonian, which appeared in different positions of the tree in different replications, and almost always outside of the Uralic node. For this reason, in the tree presented here, we placed a monophyletic constraint on the Uralic languages. An unconstrained tree is available in Supplementary Figure 8.

Apart from the Uralic issue, the main differences with UPGMA are:

(10) a. The first two splits, singling out Malagasy along with Sinitic, Japanese, Korean, and Basque^43^ from all the rest, recalling the other distance-based visualizations (Figures 1, 2).
b. The clustering of the Archi, Lak, Tamil, and Telugu node with that grouping the so-called Altaic languages and Yukaghir.
c. The reversed position of Buryat and Yukaghir.
d. The intermediate node which combines Celtic with Greek.

Differences in the sub-articulation of Germanic and Romance are discussed below (cf. Section “On the genealogical information in the syntactic trees”).

Like in the UPGMA tree, Japanese and Korean fall together, with a posterior probability of 1. Interestingly, both trees are able to assign the languages sharing some similarity in Central Eurasia (cf. Figure 1) into their different families (e.g., Indo-Iranian, Dravidian, NE-Caucasian, Uralic, Turkic).

#### Δ-Scores and Q-Residuals

A graph displaying Δ-scores and Q-residuals (Holland et al., 2002; Gray et al., 2010; Wichmann et al., 2011; Greenhill et al., 2017), along with a SplitsTree network from which they were calculated, can be found in Supplementary Material. The median of the Δ-scores is 0.302, and the variance is particularly low (standard deviation: 0.037). The 10 languages associated with the highest values (cf. Section “Network Analysis – NeighborNet” in Supplementary Material), i.e., those for which the signal is the least treelike, properly include the languages listed in (9)a, which correspond to the first two outlying branches of the BEAST tree (Mandarin, Cantonese, Korean, Japanese, the two Basque varieties, and Malagasy).

The median of Q-residuals is 0.054, but in this case the variance is quite high, in proportion (standard deviation: 0.021). Again, among the languages with the 10 highest scores, six correspond to the outliers of the BEAST tree (Malagasy has the 11th Q-residual: 0.0805). In particular, while the mean for the Δ-scores is the same as the median, the mean for the Q-residuals is higher (0.058), signaling that the distribution is skewed toward the higher values. In fact, 46 of the 69 languages show a Q-residual lower than the mean, and crucially this subset contains all the 39 Indo-European languages of the sample.

#### On the Genealogical Information in the Syntactic Trees

With few exceptions, discussed in Section “Sources of deviation”, both the UPGMA and BEAST trees capture all the taxa of our sample that are safely acknowledged by the near-unanimous judgment of historical linguists, based on lexical etymological comparison: this set will be referred to as the “Gold Standard”.^44^ Table 1 summarizes the Gold Standard nodes (second column from left), and, in the two last columns, specifies if they are captured by our UPGMA or BEAST trees. UPGMA retrieves 20/23 (87%) major families and subfamilies (21/24: 87.5%, if we include Altaic). BEAST retrieves 21/23 (91.3%) of them (or 21/24: 87.5%). Summing up, the two syntactic trees capture ∼90% of the Gold Standard.

**TABLE 1:** Our results against the Gold Standard.

	**Group**	**Languages**	**UPGMA**	**BEAST**
1	Sinitic	Mandarin, Cantonese	YES	YES
2	Dravidian	Tamil, Telugu	YES	YES
3	Basque	Basque_Central, Basque_Western	YES	YES
4	Uralic	Mari_1, Mari_2, Udmurt_1, Udmurt_2, Hungarian, Khanty_1, Khanty_2, Estonian, Finnish	YES	NO^a^
5	Altaic	Kazakh, Kirghiz, Turkish, Yakut, Uzbek, Evenki, Even_1, Even_2, Buryat	YES	NO
6	IE	Irish, Welsh, Marathi, Hindi, Pashto, Greek, Greek_Cypriot, Greek_Calabria_1, Greek_Calabria_2, Greek_Salento, Bulgarian, Serbo-Croatian, Slovenian, Polish, Russian, Faroese, Norwegian, Danish, Icelandic, German, Dutch, English, Afrikaans, French, Casalasco, Reggio_Emilia, Parma, Spanish, Portuguese, Romanian, Siciliano_Ragusa, Siciliano_Mussomeli, Salentino, Calabrese_Southern, Italian, Barese, Campano, Teramano, Calabrese_Northern	YES	YES
7	NE-Caucasian	Archi, Lak	YES	YES
8	Balto-Finnic	Estonian, Finnish	YES	YES
9	Ugric	Hungarian, Khanty_1, Khanty_2	YES	YES
10	Turkic	Kazakh, Kirghiz, Turkish, Yakut, Uzbek	YES	YES
11	Tungusic	Evenki, Even_1, Even_2	YES	YES
12	Kipchak^b^	Kazakh, Kirghiz	YES	YES
13	Celtic	Irish, Welsh	YES	YES
14	Indo-Iranian	Hindi, Marathi, Pashto	YES	YES
15	Greek	Greek, Greek_Cypriot, Greek_Calabria_1, Greek_Calabria_2, Greek_Salento	YES	YES
16	Slavic	Bulgarian, Serbo-Croatian, Slovenian, Polish, Russian	YES	YES
17	Germanic	Faroese, Norwegian, Danish, Icelandic, German, Dutch, English, Afrikaans	YES	YES
18	Romance	French, Spanish, Portuguese, Romanian, Italian, Casalasco, Parma, Reggio_Emilia, Siciliano_Ragusa, Siciliano_Mussomeli, Salentino, Calabrese_Southern, Barese, Campano, Teramano, Calabrese_Northern	YES	YES
19	Indo-Aryan	Hindi, Marathi	YES	YES
20	South-Slavic	Bulgarian, Serbo-Croatian, Slovenian	NO	NO
21	North Germanic	Faroese, Norwegian, Danish, Icelandic	NO	YES
22	West Germanic	German, Dutch, Afrikaans, English	NO	YES
23	Continental West-Germanic	German, Dutch, Afrikaans^c^	YES	YES
24	Ibero-Romance	Spanish, Portuguese	YES	YES

*^*a*^Recall that the Uralic node in the BEAST tree presented in the text is the product of an explicit constraint placed on this set of languages. ^*b*^Northwestern Turkic, Johanson and Csató (1998). ^*c*^We included the latter subfamily following Hutterer (1975, p. 195).*

## Discussion

### The Historical Signal

The results, which are consistent across all the tests performed (Heatmap, PCoA, trees), are largely at odds with statements such as Anderson and Lightfoot’s italicized quote in (1), and with the century-long assumptions behind them: syntax has provided, as a whole, a historical signal very close to that of etymological methods. We will now examine the possible roots of the deviations exhibited by syntactic parametric comparison from the expected genealogy.

#### Sources of Deviation

Deviations from the vertical historical signal can in principle be regarded as due to two factors: secondary convergence (language interference) or homoplasy (parallel independent developments produced by chance). Both are normally *a priori* removed from the input data of automatic lexical phylogenies: one wonders, then, which of these factors is really relevant to produce the deviations above. Let us focus then on the few sources of exceptions to the Gold Standard expectations as they emerge from Table 1.

The BEAST tree’s failure to capture the Uralic unity (taxon 4) is influenced by few characters in Estonian and Finnish (and their implicational consequences on some other parameters), in which these languages have a value opposite to that of the other Uralic languages and coinciding with that of all IE languages of Europe. For Estonian they are three: p15, CGB, p31, GFP, and p58, NRC, of Supplementary Figure 1. For Finnish the relevant ones are p15, CGB, again, and p32, GFN. Parameter CGB defines a macro-areal feature whose value in Balto-Finnic is shared with all IE languages of Europe, while the opposite one is shared by the rest of Uralic, the IE languages of Asia, Altaic, Caucasian, and other Asian languages. Parameter GFP has major implicational consequences on the whole Genitive system, including parameter GFN. Finally, the Estonian value of parameter NRC is the same as in all IE languages, except for some Indo-Iranian ones. These changes have assimilated Finnish and Estonian precisely to their IE neighbors, with whom very ancient loanwords have also been exchanged.^45^

Also, if an Altaic unit (taxon 5) has ever existed, a part of our experiments (cf. Figures 1, 4) expands it, by placing Yukaghir inside the supposed Altaic family. In fact, the differences of Yukaghir from Eastern Uralic are minimally more numerous than those from the Altaic languages, with which a century-long situation of bilingualism/diglossia as a lingua franca in NE Siberia is well documented.^46^

The outlying position of Bulgarian in both trees (which fail to capture the South Slavic unity, taxon 20) can be traced to relatively recent horizontal parametric convergence; in particular, there are two relevant parametric differences making Bulgarian slightly eccentric with respect to the rest of Slavic:^47^ Bulgarian is the only Slavic language (with Macedonian) which selects the value “+” for p17, DGR, like its neighbors Romanian and Greek (it has developed a definite article, and indeed an enclitic one, like Romanian: p24, DCN^48^), and has developed a prepositional Genitive/Dative, like Romanian (cf. p41, GAD).^49^ These have long been considered among the areal features of the Balkans.^50^ So-called Old Bulgarian (Old Church Slavonic) had the value “−” for DGR. Notice also that DGR starts a long sequence of implications, so that its “−” setting in other Slavic languages *a priori* neutralizes a large number of potential similarities with Bulgarian.

Finally, the UPGMA tree fails to identify West Germanic (taxon 22). As a matter of fact, issues concerning the internal classification of Germanic have been acknowledged in all the quantitative literature.^51^ In particular English (along with Afrikaans) has historically experienced most contacts with other Germanic and non-Germanic languages. Furthermore, English has also been recently the focus of a debate between Emonds and Faarlund (2014) and their reviewers and critics^52^ about whether, from the Middle English period on, it must be considered a prevailingly Scandinavian rather than West-Germanic offspring (if not the continuation of a creolized version of the two). The unstable position in our experiments confirms that the question is at least a meaningful one. Anyway, it is a fact that English was in close contact with Nordic tribes in both its prehistoric^53^ and historic dwelling areas.

In all the cases above, two properties hold: (i) the syntactic detachment of a language from a traditionally expected position in the tree correlates with exhibiting similarity with some neighboring languages; (ii) these deviations from the Gold Standard appear to always be tied to situations of horizontal transmission independently witnessed by other linguistic levels.^54^ This confirms Thomason and Kaufman’s (1988) conclusion that syntactic borrowing takes place in conditions of “intense” contact, quantitatively measurable by other linguistic variables.

Given the binary nature of our syntactic characters, as opposed to the virtually infinite possibilities provided by lexical arbitrariness, one might think that homoplasy (hence accidental failure of the signal) plays the main role in the deviations from the Gold Standard. On the contrary, the picture suggests that the differences between the syntax trees and the accepted lexical wisdom are always imputable to interference (itself a historical factor), and do not necessarily call for the intervention of homoplasy.

#### Vertical and Horizontal Transmission

Even horizontal effects have relatively little impact on the general topology of the tree. For instance, under all our experiments, the Italiot Greek varieties cluster with Standard and Cypriot Greek: the protracted contact and documented syntactic interference between Romance and Greek in Southern Italy^55^ have not disrupted the overall vertical signal of either family. To measure the conflict between vertical and horizontal information in the signal, we used Δ-scores and Q-residuals. Recall that a lower value of these indices speaks for a sharper vertical signal.

Δ-scores in our experiment, with a median as low as 0.302, yield better results than those obtained in both datasets used in Greenhill et al. (2017), where lexical characters displayed a median of 0.38 and structural characters displayed one of 0.44.

The Q-residuals perform less well: Greenhill et al. (2017) had a median of 0.0062 for lexical characters and 0.0354 for structural characters, against our median of 0.054.^56^ Notice, however, that Wichmann et al. (2011) tested the two measures on a group of languages of the Automatic Similarity Judgment Program database,^57^ and noticed that Δ-scores distributed uniformly with respect to age and size of the language family; Q-residuals instead correlated with such factors, becoming higher and less informative for chronologically deep and numerous and internally diverse families. Based on these results, they argued precisely in favor of Δ-scores as more accurate measures of non-tree-likeness. This seems to be true in our experiment as well: the highest Q-residuals are associated with languages occurring on the higher branches, whose genetic affiliation is still unclear; but all Indo-European languages display Q-residuals lower than the mean, suggesting that the measure is indeed sensitive to the age and size or diversity of the family (cf. Section “Δ-Scores and Q-Residuals”). This is not true for Δ-scores: while the outliers equally display high Δ-scores, IE languages are more evenly distributed above and below the mean (23 vs. 16). If Wichmann et al. (2011) are right, then, our result is expected: it is likely that Q-residuals cannot meaningfully apply to long-range classifications across many different families.

### Ultralocality: Hints About Microvariation

The internal articulation of the Romance dialects of Italy retrieved by the UPGMA tree is consistent with their traditional classification.^58^ The tree clusters them together, then identifies the Gallo-Italic group (Reggio Emilia, Parma, and Casalasco), the Extreme southern group (Siciliano, Southern Calabrese, and Salentino), and one that clusters three Upper southern dialects (Campano, Teramano, and Barese) but not Northern Calabrese: this may reflect the isolation of this dialect as representative of an area known to exhibit several peculiarities with respect to the whole Italian group.^59^

At this level of microvariation, no taxonomy can be really projected onto a genuine phylogeny, because of the uninterrupted contact and diffusion of isoglosses among contiguous dialects (cf. the network and the PCoA in Supplementary Figures 14, 16; also cf. Sarno et al., 2014 on strong genetic admixture in Southern Italy). This may have produced the differences between the UPGMA and BEAST trees: the BEAST tree may rather highlight the actual secondary relations which have occurred between Sicilian and Ibero-Romance, some closeness between Gallo-Italic and French, and also plausible interference of Balkan languages with Salentino, which appears as the outlier of all of Romance.

Thus, even minimally different character strings and very short parametric distances have good resolution power. Moreover, the fact that parametric distances become very low at this level of comparison is exactly what we expect if syntax evolves proportionally to other historical variables.

The resolution we obtain in micro-variation is inevitably based on parameters which must have undergone recent changes, i.e., which, virtually by definition, are not as stable as others. Yet, their instability has not produced any conceivable disruption of the correct topology in other areas of the phylogenies. This very consequential observation is discussed in Section “Input data and phylogenetic results”.

### Globality: Hints About Long-Range Relations

The most salient feature of parametric systems is their potential universality. Accordingly, our phylogenetic analyses provide some preliminary insights about possible or proposed long-range groupings. They will eventually have to be evaluated through more elaborate statistical analyses, but provide a list of heuristic suggestions for further testing.

First, nearly all the experiments single out a set of languages as outlying the rest of the sample: Japanese, Korean, the two Sinitic and two Basque varieties, and, except for the UPGMA tree, Malagasy. The other languages are always identified as a mono-phyletic structure and Δ-scores and Q-residuals suggest that they have a more reliable vertical articulation.

In addition to recognizing all classical families, our data suggest that Indo-Iranian, Dravidian, NE-Caucasian, Turkic, Tungusic, Buryat, Yukaghir, and part of Uralic partake of some similarity, which is especially highlighted in Figure 1; however, such similarity turns out to be weaker than the respective family affiliations (cf. the trees in Figures 3, 4). The methods used cannot decide how much of this similarity is secondary and areal, though the fact that (only) the IE languages of Asia share it, and (only) the Uralic languages that dwell in Central-Western Europe (Hungarian, Finnish, Estonian) do not, suggests that part of it must be.

Next, all experiments point to the unity of part of the controversial Altaic family (Turkic and Tungusic), and a weaker connection of this cluster to Buryat (Mongol), but also to Yukaghir.

Even more robustly, the syntactic analysis argues for a Korean-Japanese relation, although sustained by a relatively low number of non-null comparisons (30 pairs; only 12, according to a Jaccard measure). Statistical support is very high, as is only the case, in our sample, for a few safely established pairs/groups. Notice that some studies have proposed that even sound correspondences support the relatedness of Japanese and Korean.^60^

Notice, instead, that the clustering of Korean and Japanese with Mandarin and Cantonese in both trees should not deceive us, because it is likely to be a bias of the tree algorithms (clustering together data points which are both outliers with respect to the main group of taxa is a common error, usually described as Long-Branch Attraction: Bergsten, 2005). This becomes clear from the distance distribution: in Figure 1, the two groups are clearly set apart; moreover, if we draw a PCoA specifically focused on the languages of the upper left quadrant of Figure 2, Japanese-Korean and the two Chinese varieties clearly fall into distinct quadrants (cf. Supplementary Figure 3).

Finally, none of our experiments hints at a Macro-Altaic grouping.^61^ However, the syntactic data cannot exclude some genealogical relation between Korean-Japanese and central Asian languages, with secondary influences from the East Asian area.^62^

A worth exploring relation is that between Uralic and Altaic. Uralic languages are scattered in terms of distance but, with the exception of Balto-Finnic in the BEAST tree, they are recognized as a unit. In spite of the noted similarities with IE languages, the syntactic data provide sufficient evidence that Balto-Finnic is indeed a Finno-Ugric family influenced by IE rather than the opposite, and that, if anything, the whole Uralic is closer to Altaic than to Indo-European. First, when we place a monophyletic constraint on the set of Uralic languages in the BEAST phylogeny, the stable result is that Uralic is clustered with the Altaic-Yukaghir node. Second, the other Uralic languages are never separated from the Altaic group in any experiments (cf. Section “Phylogenetic Analysis – BEAST 2” in Supplementary Material). Third, the Genitive systems of Estonian and Finnish (and the pronominal possessive system of Estonian), which oppose them to all the other Uralic (but also Turkic and Tungusic) languages (cf. Section “Sources of deviation”), must be regarded as an innovation with respect to the others: it has involved the loss of agreement between the features of a Genitive and those expressed through a dedicated morpheme on the head noun, a common Uralic feature.^63^ The weakening or loss of such morphemes is a well-known diachronic phenomenon, attested, e.g., for verbs and adjectives in the history of Romance and Germanic (possibly an instance of what Keenan, 2009 considers phonological “DECAY”); its creation anew is not easily observed. All this is consistent with the possibility of some Uralo-Altaic unity, blurred by the Indo-Europeanization of the Balto-Finnic languages, while it makes any original Indo-Uralic unity excluding Altaic and Yukaghir highly unlikely.^64^

All experiments also point to significant closeness of NE-Caucasian and Dravidian (average distance 0.23). This similarity, which needs to be investigated, connects to another stable outcome of our experiments: the fact that Basque lies outside the group constituted by the other Eurasian languages except for those of the Far East, and, in particular, does not show any trace of the sometimes proposed relation to the NE-Caucasian languages (average distance 0.51).^65^

### The Homology Conjecture

We conclude that (A) syntactic phylogenies are very similar to the lexical-etymological ones, and (B) the small proportion of deviation can be imputed to secondary convergence only (which so far has been *a priori* removed from lexical, though not syntactic, data). These two claims are merged into:

(11) The **Homology Conjecture:** Syntactic and lexical histories provide the same evolutionary topologies once interference is equally taken into account

This hypothesis is in agreement with the expectations of syntactic *Inertia* (cf. Section “Syntax, Cognitive Science, and Historical Taxonomy”) and is parallel to the Neogrammarian Regularity hypothesis, in attributing any disruption of an ideal diachronic evolution (in that case, regularity of non-analogical sound change) to dialect admixture.

### A Comparison With Phonemic Inventories

We checked then what kind of signal can be retrieved from our language sample through non-lexical (and potentially cross-family) traits that are not characterized by the three formal properties we used to select our syntactic characters (cf. (3)), and that are more remote from the core generative mechanisms of grammar.

**FIGURE 5:**
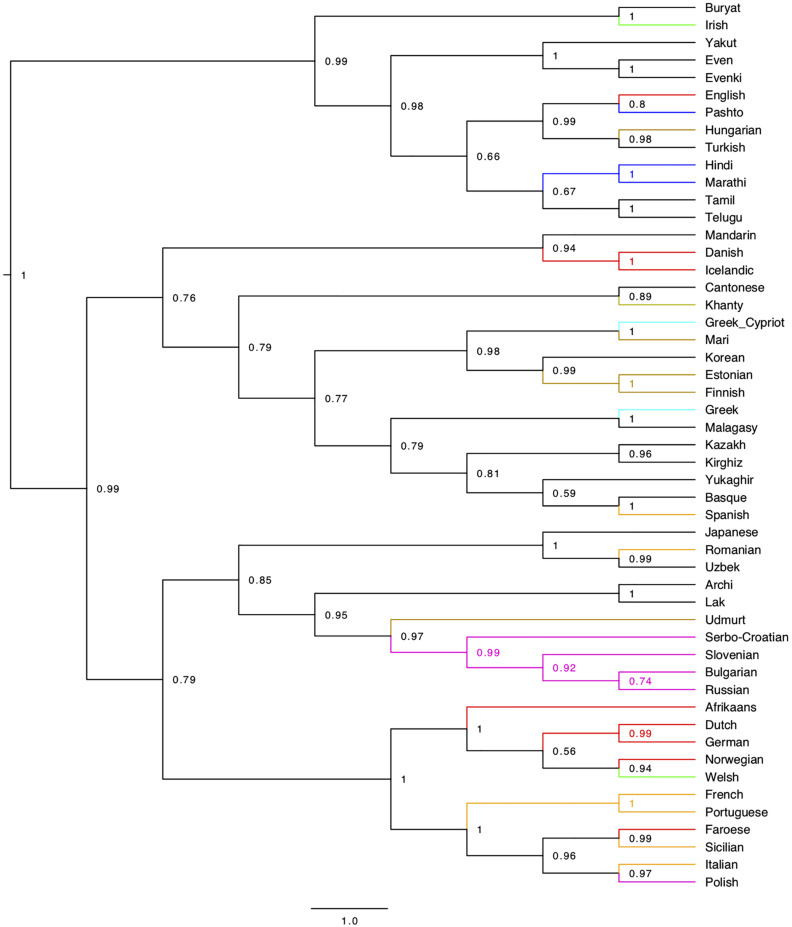
BEAST tree from Ruhlen’s phonemic dataset. The tree contains a subset of the languages used in Creanza et al. (2015), consisting of the 52 languages overlapping with those used in this article. The color coding is the same as for the previous phylogenies, visually highlighting the differences in the clustering of the families. The best model that we determined is a Gamma Site Model with Substitution Rate = 1, a Mutation Death Model with death *p* = 0.1, a Relaxed Clock (Logarithmic) with clock rate = 1, and a uniform Yule model for the birth rate. The Monte Carlo Markov Chain produced 10,000,000 trees, 25% of which were used for the burn-in and discarded for the purpose of the calculation of the consensus tree. The tree is a consensus tree of 7500 different trees sampled through the 7,500,000 trees (with a sample stored every 1000 generated trees) produced by the Monte Carlo procedure.

For instance, inventories of autonomous phonemes have been used for comparison across different families, e.g., in Creanza et al. (2015). This work employs two large phonemic databases, PHOIBLE^66^ and Ruhlen,^67^ in an attempt to align phonemes into corresponding classes based on phonetic similarity.^68^ To check whether phonemic characters generate informative phylogenies at our scale/density of sampling, we generated a BEAST tree (Figure 5) from the entries in Ruhlen’s data corresponding to the languages of our study. The only taxa of the Gold Standard above identified by this tree are the 5 (21.7%) listed in (12):^69^

(12) a. Dravidian
b. Indo-Aryan
c. Tungusic (Even, Evenki)
d. Balto-Finnic
e. NE-Caucasian

These pairs are also geographically close and might in part reflect reciprocal secondary influence, as the cluster Spanish/Basque apparently does. Most other clusters do not reflect historical information at all (e.g., Sicilian-Faroese, English-Pashto, Irish-Buryat, Mari-Cypriot Greek etc.).

Our experiment supports Creanza et al.’s (2015, p. 1269) claim that “phoneme inventories are affected by recent population processes and thus carry little information about the distant past”:^70^ phonemic data exhibit a much shallower historical signal than syntactic data, and are actually prone to detect secondary convergence (see also Wichmann and Holman, 2009). This result shows the relevance of comparing different input data and prompts some considerations about their historical signal.

### Input Data and Phylogenetic Results

Some previous phylogenetic experiments found less historical signal when looking at structural traits. For instance, Greenhill et al. (2017) compared the evolutionary rate and signal of lexical etymologies with that of some structural properties in 81 Austronesian languages. They found that, on average, structural properties display higher rates of change than lexical sets, and that there are subsets of properties (both lexical and structural) that change much slower or much faster than the average. For instance, number marking on the noun phrase and the presence of tones showed up as conservative, while article properties and vowel length as features that tend to change over time.

Thus, in certain respects, the historical signal retrieved through the syntactic dataset of the present article is more robust and promising than that obtained with their structural traits: the results are not necessarily in contrast, though, because of the different properties of the input data and of the different idealizations made on them (cf. (3a–c)) in Section “Syntactic data and taxonomic problems”.

First, one difference is that the structural traits used in Greenhill et al. (2017), like those employed in a preliminary work by Dunn et al. (2005), include not just syntactic characters but also other non-lexical features, some of which (presence of phonetically defined autonomous phonemes) are shown here to contain a shallow and genealogically very disruptive signal. So, this is a potential cause of the different outcome.

Second, parameters are coded as representations of the generative devices in mental grammars, rather than as generated patterns. It is conceivable that this provides them with a high degree of cognitive realism and deductive information, which in turn provide historical resolution. Recall that only an average of 20 parameters (39 if we consider identities on the “−” values) are fully comparable across the language pairs of our sample, due to the redundancies created by the pervasive implicational structure of parameters (cf. Section “Parameters and schemata”). The correctness of the topologies retrieved by so few characters suggests indeed that parameters do have high-resolution.

Finally, a most interesting property brought to light by our experiments is that all the divergences of syntax from the established or expected topologies can in principle be explained in terms of secondary convergence: neither of the syntactic topologies presents clear cases where an incorrect cluster is exclusively determined by homoplasy. Notice that *a priori* we might expect homoplasy to seriously affect syntactic topologies, given that our characters are binary and that we deal with many independent families. However, this is not the case. This may in part be due to the general robustness of the complementary vertical signal; but a relevant role must be played here by the third property of parametric data, their pervasive interdependence: the redundancy provided by parametric implications neutralizes the effects of the most obvious source of homoplasy. The resolution we obtain in the articulation of families and subfamilies, up to recently and minimally differentiated dialects, comes at the cost of considering at least some traits with a high-rate-of-change, which discriminate between close varieties; thus, by definition, they are less stable than parameters that have remained unchanged for millennia all over large families. In principle, their instability might have produced a great amount of homoplasy elsewhere in the trees, disrupting the correct phylogenies across other families. Yet, this has not happened with our dataset. Many parameters in Supplementary Figure 1 which make finer distinctions within Romance dialects (and other close varieties) are neutralized in most non-Romance (or non-IE) languages, due to their dependence on hierarchically higher parameters. This has reduced accidental similarities between distant families. It is plausible that any attempt to attain globality with grammatical characters, in order not to crash against homoplastic effects, must indeed take into account the pervasive interdependence of such traits.

## Conclusion

Five major inferences can be drawn from the results of this article.

### The Historical Signal of Syntax

The syntactic structures of I-languages (Chomsky, 1986: the abstract rule systems of computational theories of mind; also see Everaert et al., 2015) are an effective tool of historical knowledge (*pace* contrary positions in comparative philology and in modern formal syntax, as well as some skepticism expressed in quantitative phylogenetics: cf. Dunn et al., 2011): they retrieve most of the phylogenetic information contained in trees produced by lexical etymologies. Strikingly, the trees obtained from syntax are essentially unaffected by the inevitable amount of homoplasy which must be produced by the binary nature of the characters used. Also, the verticality of the syntactic signal and its chronological depth are far stronger than those of more externalized traits, like phonetic similarity in phonemic inventories (in agreement with Creanza et al.’s, 2015 conclusion that such phonemic characters are not informative about deep-time relations). The phylogenies retrieved through syntax must be relatively deep in time, if they are able to sharply separate, e.g., Basque from IE and other Eurasian families: given the limitations of (non-speculative) methods for investigating deeper language evolution, stressed in Hauser et al. (2014), this empirical, bottom-up approach is a promising perspective for studying the past of human syntax.

### Historical Support for Generative Grammars

The search for a historical signal represents an unprecedented type of evidence to test the format of representation of mental grammars used in syntactic theories, especially in minimalist approaches to parameters. As in the formal grammatical tradition, we have tried to model the dataset used not simply as a set of experiential facts, but mostly as a deductive structure in which surface data (e.g., E-languages) are largely the product of the combination of simpler and less numerous principles (I-languages). The success in retrieving a historical signal corroborates this general approach on a domain different from the usual ones (synchrony, typology, acquisition) used to support formal linguistic theory.

### Generative Grammars and Phylogenetic Evidence

Conversely, this robust historical signal suggests a reconsideration of the practice of formal syntax itself: for example, when a clear deviation of a parameter value occurs in a language from the state of its established family, it will call for an explanation. If the synchronic analysis is correct, then for linguistic theory the question should arise of how, and possibly why, the disconnection from the family pattern has taken place.

### Phylogenetics and Language Distances

Beyond some minor complementarity between character- and distance-based models of syntactic history, the topologies retrieved by the two methods are quite similar. This is in line with Greenberg’s (1987) controversial claim that a first approximation to language taxonomy is possible even ahead of step-by-step reconstruction of all ancestral characters.

### Tools and Perspectives

We have used a tool for language description (a list of YES/NO existential questions: cf. Crisma et al., 2020) universally applicable and requiring very limited information (in principle no more than one YES answer per parameter set to “+”): this was mainly possible owing to the redundancy and default settings which characterize a minimalist approach to parameters. Beyond phylogenetics, a system with these properties has obvious consequences for the study of grammatical diversity and language learnability (cf. Sakas et al., 2017).

In sum, we regard these results as a breakthrough with respect to a long tradition in linguistics: they indicate that there exists a signal in syntax which might be used for aiming at progressively more comprehensive phylogenies of human languages. We suggest the possibility of adding less visible taxonomic traits, such as syntactic parameters, to the toolkit of phylogenetic linguistics as the basis for a *qualitative* revolution, which may complement the scope and success of the *quantitative* one.

## Data Availability Statement

The code used to generate the experiments and the figures can be found at https://github.com/AndreaCeolin/FormalSyntax doi: 10.5281/zenodo.4323165.

## Author Contributions

GL and CG devised the comparative methodology and the specific parametric structure. GL, CG, MAI, and AC collected the data. AC performed the computational experiments. GL, MAI, and AC wrote the Introduction. GL, CG, and AC wrote the Materials and Methods, the Results, and the Discussion. GL wrote the Conclusion.

## Conflict of Interest

The authors declare that the research was conducted in the absence of any commercial or financial relationships that could be construed as a potential conflict of interest.

## Footnotes

1 Ringe et al. (2002); Gray and Atkinson (2003); McMahon and McMahon (2005); McMahon (2010), and the stream of subsequent work.

2 E.g., Braudel (1958) and subsequent work, Diamond (1997); Smail (2008).

3 Cavalli-Sforza et al. (1994), as well as subsequent work.

4 Cf. Hauser et al. (2002); Boeckx and Piattelli-Palmarini (2005); Di Sciullo and Boeckx (2011); Berwick and Chomsky (2015), a.o.; for some specific applications to language diversity see Biberauer (2008); Karimi and Piattelli-Palmarini (2017); Roberts (2019) and much cited literature.

5 E.g., Kraus (1787; see Kaltz, 1985 for details), Adelung (1806–1817) or Balbi (1826a,b). “Balbi (1826a, xlii f., note) … stated that grammatical comparison cannot be used to establish kinship and quoted as an example the fact that English and Omagua, a language of Brazil, were clearly not related, though their grammars contrasted in similar ways with the grammars of other languages in their families (ibid., 28).” (Morpurgo-Davies, 1992/2014, p. 51).

6 Morpurgo-Davies (1992/2014) points out that even Hervás (1778–1787, 1800–1805) or Gyarmathi (1799), though interested in grammatical features, did not go beyond the examination of traits such as declensions, conjugations, degrees of comparison of adjectives, suffixes used to mark comparable functions, pronouns, etc., i.e., the lexically arbitrary coding of form-meaning in functional elements. She notices that later and more established names in comparative reconstruction (Schleicher, for example) equally considered that only phonology and morphology were relevant for historically oriented work.

7 After the programmatic concepts in Klima (1964, 1965), the question of the potential of grammatical features for historical relatedness was not fully resumed until Nichols (1992); Longobardi (2003); Dunn et al. (2005); Guardiano and Longobardi (2005); Wichmann and Saunders (2007), and a first systematic use of formal syntactic traits was only attempted in Longobardi and Guardiano (2009). An interesting exception regarding syntax as an indicator of relatedness is Chapin (1974), kindly pointed out to us by R. Kayne.

8 It is normally assumed to be like further features of language and culture, and unlike certain other cognitive faculties (there is a sense to the notion “French syntax,” no less than to “French vocabulary,” or “French cuisine,” though not to “French memory” or “French visual perception”).

9 All this foundational work of Lightfoot’s on diachronic syntax, as well as that inspired by Kroch (1989 and subsequent: especially see Pintzuk and Kroch, 1995 on dating) has not been concerned with relatedness, as noted. Nonetheless, this line, along with Kayne’s (1975, 2000 and subsequent) insights on comparative syntax, has been essential for conceiving of generative grammars as tools of historical knowledge.

10 In fact, there have been sporadic, though insightful, suggestions that syntax may be even more conservative than other linguistic levels, at least as a source of primitive diachronic change. This is basically the content of Keenan’s (2002, 2009) notion of Inertia, i.e., the hypothesis that linguistic structure tends to stay stable through time “unless acted upon by an outside force or DECAY” (Keenan, 2009, p. 18). “Decay” here refers to phonological erosion and lexical-semantic impoverishment. A slightly more articulated definition of the Inertia hypothesis has been adopted in Longobardi (2001): “…syntactic change should not arise, unless it can be shown to be *caused*—that is, to be a well-motivated consequence of other types of change (phonological changes and semantic changes, including the appearance/disappearance of whole lexical items) or, recursively, of other syntactic changes…” (Longobardi, 2001, p. 278).

11 For progress in the automatization of lexical comparative methods also see List (2014).

12 Crucially, we do not use nano-parameters, which involve extensional definitions in terms of lists of lexical items.

13 See Longobardi and Guardiano (2009) for an extensive justification of these methodological assumptions.

14 In the terms of early modern biology.

15 See especially Boeckx and Leivadá (2014); Fodor and Sakas (2017); Lightfoot (2017), and the various problems summed up in Longobardi (2017).

16 Rizzi (1978, 1982); Taraldsen (1980); Chomsky (1981); Kroch (1989); Kayne (2000), a.o.

17 Several parameters concerning the Determiner category and Genitive Case used in this article are analyzed in syntactic detail in Crisma and Longobardi (in press) and in Crisma et al. (to appear). Notice, however, that, in order to conform to the requirement that the “+” state must be settable on the basis of positive evidence only, the formulation of some parameters here can have reversed the “−” and “+” values (see Crisma et al., 2020).

18 Considering Turkic, Mongolic, Tungusic, Japanese, and Korean as separate families, since there is no consensus in the field about their genealogical relatedness (see e.g., Ceolin, 2019).

19 The Italo-Romance dialects of our sample belong to three major groups (Pellegrini, 1977; Loporcaro, 2009): (1) Gallo-Italic: Casalasco (Vezzosi, 2019), Reggio Emilia, Parma. (2) Extreme southern: Reggio Calabria (Southern Calabria dialects are usually clustered with Sicilian dialects), Salentino (traditionally classified as an Extreme southern dialect but geographically separated from the rest of the Extreme group, while it has enjoyed an uninterrupted road connection to Rome and Naples since the Via Appia was built between 312 and 264 BC), two dialects from Sicily (Ragusa and Mussomeli; see Guardiano et al., 2016). (3) Upper southern: Teramano, Campano, Barese, and Northern Calabrese. The latter belongs to a particularly conservative area (Lausberg, 1939) characterized by morpho-phonological features which single it out from the rest of Italian dialects (Rohlfs, 1972; Rensch, 1973; Fanciullo, 1988, 1997; Martino, 1991; Romito et al., 1996, a.o. and also Silvestri, 2013 and Guardiano et al., 2016 about its nominal syntax).

20 In the Greek group, we selected the following varieties: Standard Modern Greek, Cypriot Greek, and three varieties of Italiot Greek (one from Salento and two from Calabria which display different degrees of conservativity, Guardiano and Stavrou, 2014, 2019, 2020; Guardiano et al., 2016).

21 Also see Baker (2001); Roberts (2019), a.o.

22 This figure goes down to 20 if only “+/+” is computed as an identity: cf. footnote 29.

23 The language that has the highest amount of “+” is Romanian (29), while the language with the smallest amount of “+” is Cantonese (9).

24 Eisen et al. (1998); Cordoni et al. (2016).

25 Davis (1986); Podani and Miklos (2002).

26 Sneath and Sokal (1973).

27 Saitou and Nei (1987).

28 Rigon (2009); Longobardi et al. (2013).

29 The average number of parameters that are comparable in our dataset according to the Jaccard metric (i.e., parameters where either language displays a “+” without the other displaying a “0”) turned out to be 20, with a range between 7 and 30.

30 Cf. Franzoi et al. (2020) for an attempt to develop metrics alternative to Hamming and Jaccard in order to capture structural dependencies among characters. Their work interestingly shows that variation in the choice of distance formulae produces limited perturbations of the robustness of the signal when applied to syntactic data.

31 Cf. Swofford (2001); Schmidt et al. (2002); Ronquist and Huelsenbeck (2003); Yang (2007); Drummond and Rambaut (2007); Tamura et al. (2011); Rambaut et al. (2018), a.o.

32 E.g., Gray and Atkinson (2003); Bouckaert et al. (2012); Chang et al. (2015).

33 Gray et al. (2010); Greenhill et al. (2017).

34 Bryant and Moulton (2004).

35 So coding “0” would force the method to postulate multiple changes when in fact a single one occurs, and in many cases this would lead the algorithm to reconstruct the wrong node for a certain group, and then spreading the error through the tree.

36 Note that this does not prevent the algorithm from considering and sometimes selecting reconstructions of ancestral states incompatible with the implicational structure of the dataset.

37 Roberts and Roussou (2003); Heine and Kuteva (2005).

38 More specifically Finno-Ugric, represented by two Balto-Finnic languages, three Ugric varieties, two Udmurt (Permic) and two Mari (Volgaic) ones.

39 Kazakh and Kirghiz (Kipchak sub-branch, Northwestern Turkic, Johanson and Csató, 1998); Turkish (Oghuz sub-branch, Southwestern Turkic: Menges, 1968; Schönig, 1997–1998, a.o); Uzbek (Karluk sub-branch, Southeastern Turkic, Schönig, 1997–1998, a.o.); Yakut (Northeastern Turkic).

40 Ewenic: Evenki, Even1, and Even2, Khabtagaeva (2018), a.o.

41 Vovin (2005); Robbeets (2005); but also see Doerfer (1985); Tekin (1994); Soucek (2000); Shimunek (2017), a.o.

42 There are no other Austronesian languages in our sample.

43 I.e., the languages of the upper left quadrant of Figure 2 above.

44 This is the most reliable procedure to evaluate the results of a phylogenetic analysis (cf. Greenhill et al., 2020). From the Gold Standard set we excluded the possible clusters of the micro-variation level, throughout all the families, since their identification in traditional literature is often based on non-vertical evidence and involves geographical and sociolinguistic considerations.

45 Kylstra et al. (1991) suggest that the first contacts between Germanic and Balto-Finnic date from around 1000 BC.

46 Wurm et al. (2011, pp. 970, 978).

47 Cf. Longobardi et al. (2013).

48 For this circum-Pontic isogloss see Guardiano et al. (2016).

49 This, in turn, may have enabled the resetting of p43, GFO, as well, i.e., the disappearing of an inflected Genitive.

50 See Sandfeld (1930) and now, specifically for syntactic borrowing, Tomić (2006).

51 Dyen et al. (1992); Ringe et al. (2002); Jäger (2015).

52 Barnes (2016); Bech and Walkden (2016); Stenbrenden (2016); Crisma and Pintzuk (2019), and the contributions to the 2016 issue, 6.1, of *Language Dynamics and Change*.

53 Hutterer (1975), a.o.

54 Even the internal comparison between the UPGMA and the BEAST trees turns out to be informative to confirm cases where the signal is conflicting, i.e., one or more languages can be associated with different phylogenetic histories.

55 Guardiano and Stavrou (2014, 2019, 2020); Guardiano et al. (2016); Ledgeway (2006); Ledgeway (2013); Ledgeway et al. (2018), a.o.

56 Greenhill and his collaborators (p.c.) suggest that this difference can be explained as a result of the fact that while Δ-scores might be more sensitive to conflicting signal (i.e., the presence of two alternative histories for a taxon), Q-residuals might be more sensitive to noise in the data.

57 ASJP, Wichmann et al. (2020).

58 Pellegrini (1977).

59 Lausberg (1939).

60 For instance, Whitman (2012); also see the discussion in Robbeets (2008a), a.o.

61 Altaic-Korean-Japanese: see the discussion in Port et al. (2019) and the Trans-Eurasian hypothesis of Robbeets (2008b).

62 The consequence of such influences is reasonably the degrammaticalization of Person and Number features (p5 FGP and p7 FGN), which are rich in neutralizing implicational effects on further parameters. Indeed, after close consideration of the parameter values, the 0s induced by the lack of value “+” for FGP is the main source of peculiar similarity between Mandarin-Cantonese and Korean-Japanese. Beyond this, the parameters in which the four languages share a value in contrast to all the other languages are only two: p27, FGE, about the necessity of a classifier between a numeral and a head noun (itself a property very frequent in languages without a positive value at FGN: see Cathcart et al., 2020), and p61, LKP, about the presence of a special morpheme linking the noun with essentially any of its arguments.

63 Collinder (1960).

64 Also see Marcantonio (2002) and the debate ensued.

65 Starostin (1996) and Bengtson (2017), a.o.

66 Moran and McCloy (2019).

67 http://starling.rinet.ru/typology.pdf

68 Of course, it is plausible that an interesting historical signal can be retrieved from analyses of more abstract phonological processes and constraints rather than just of the physical resemblance of autonomous phonemes. Promising results on this line, which parallel the ones of our approach, are provided in Macklin-Cordes et al. (2020).

69 Few other clusters with more indirect genealogical content are those formed by two continental West Germanic languages (German and Dutch), two Northern Germanic languages (Danish, Icelandic), four Slavic languages (Bulgarian, Russian, Slovenian, Serbo-Croatian), and two Romance languages (Portuguese and French).

70 Creanza et al. (2015) complement this claim with pointing out the limited and historically recent correlations found between phonemic distances and genetic distances. Using syntactic parameters, instead, Longobardi et al. (2015) found that genetic differences correlate with linguistic distances more than with geographic distances in Europe.
