# Supplementary figures and images for: Formal Syntax and Deep History
Source: Front Psychol. 2020 Dec 18;11:488871. doi: 10.3389/fpsyg.2020.488871 (PMC7775603; doi:10.3389/fpsyg.2020.488871)

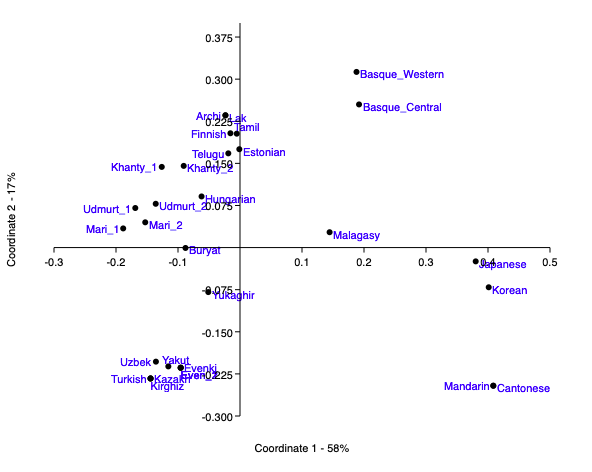

Supplement: Supplementary file 3 [file Image_2.png]

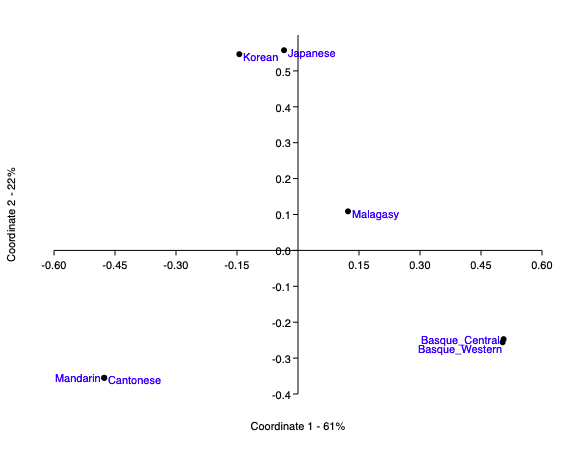

Supplement: Supplementary file 4 [file Image_3.png]

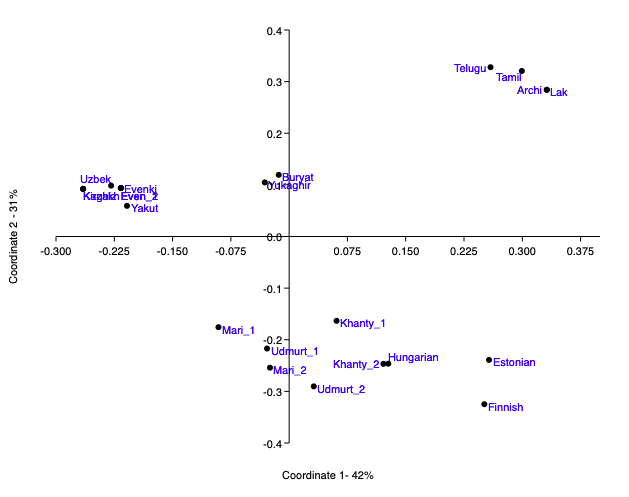

Supplement: Supplementary file 5 [file Image_4.png]

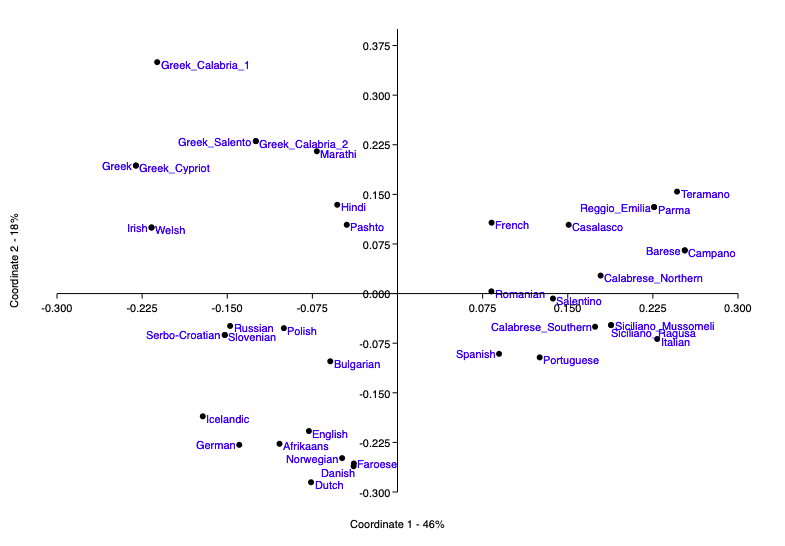

Supplement: Supplementary file 6 [file Image_5.png]

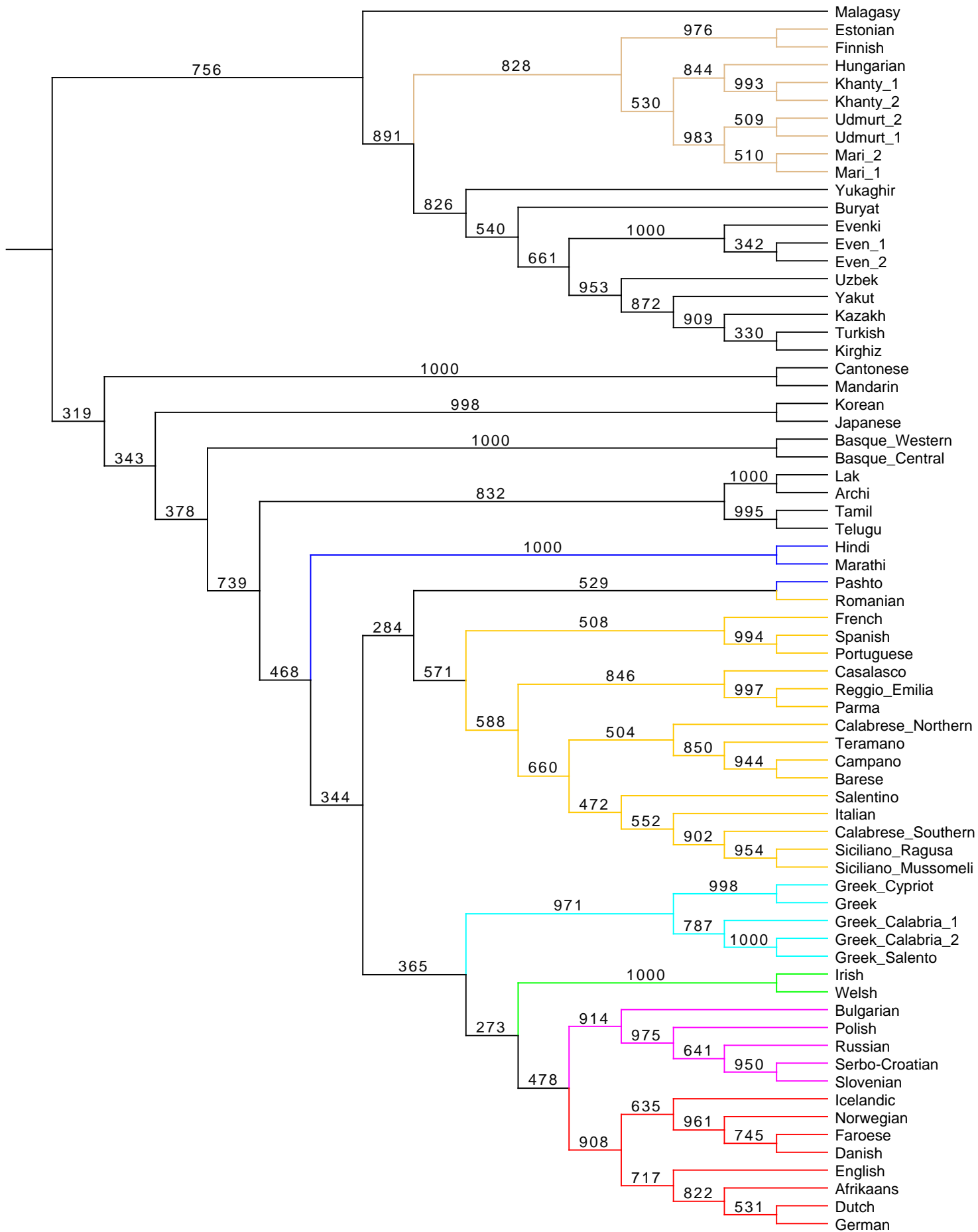

Supplement: Supplementary file 7 [file Image_6.pdf]

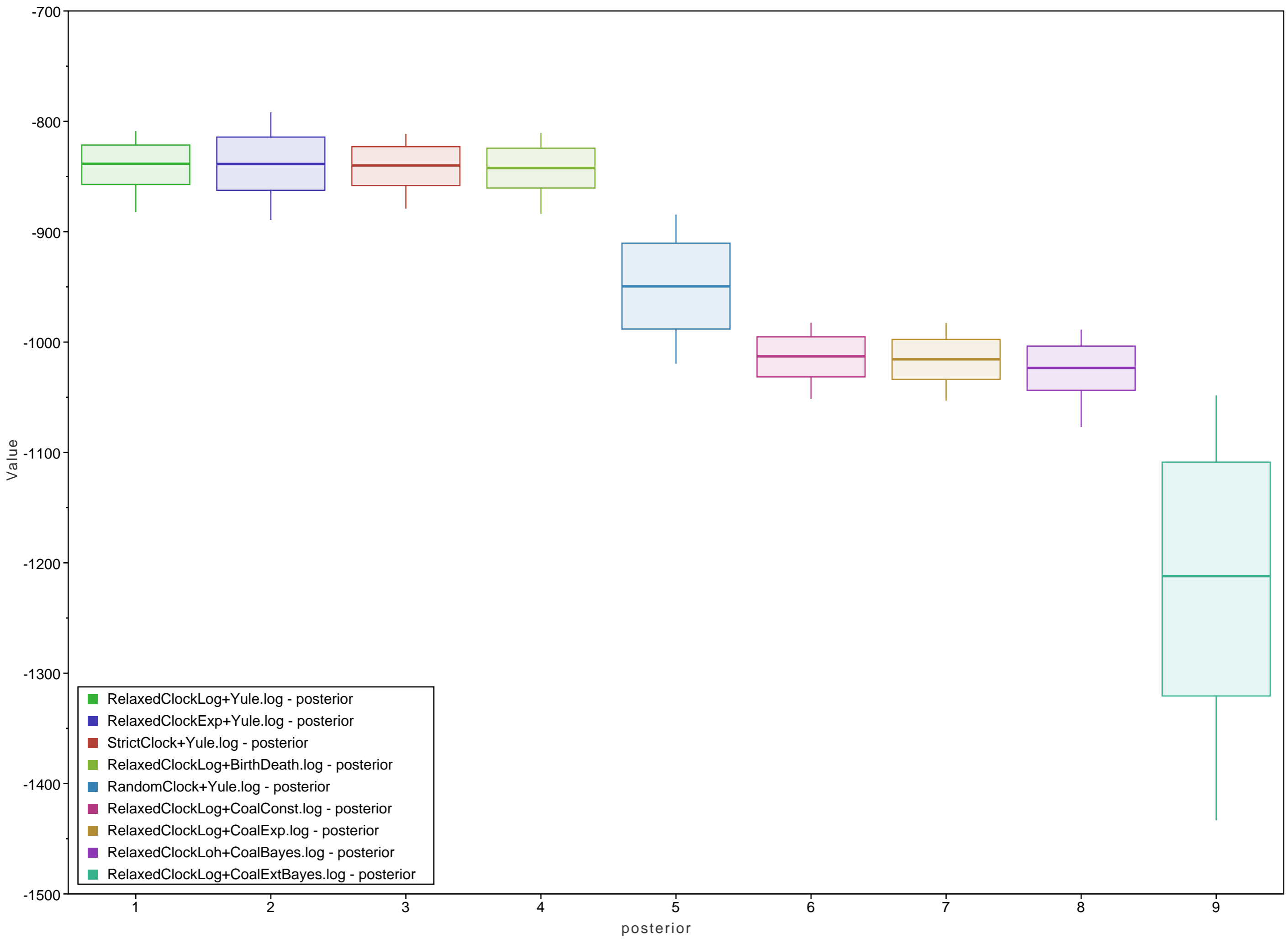

Supplement: Supplementary file 8 [file Image_7.pdf]

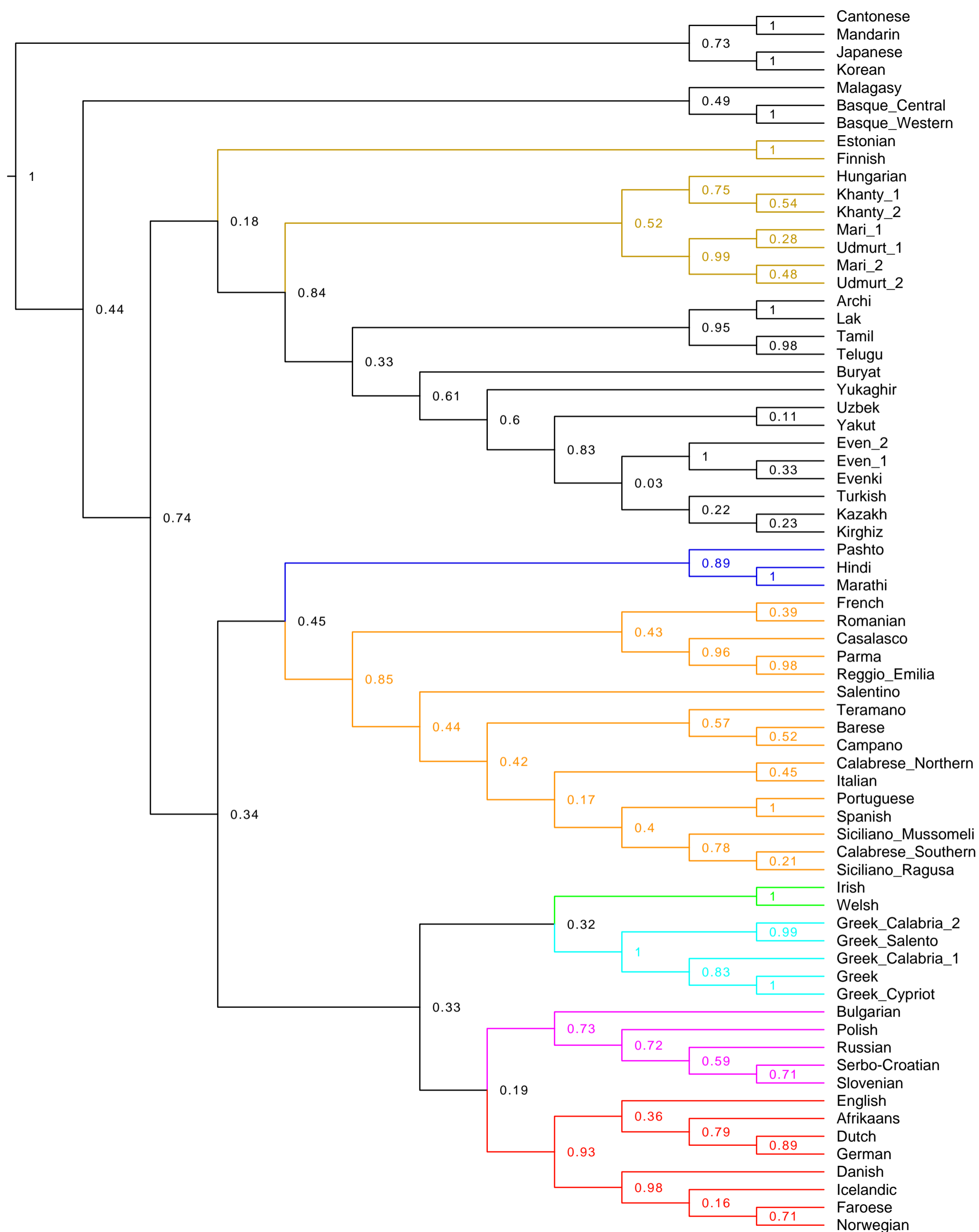

Supplement: Supplementary file 9 [file Image_8.pdf]

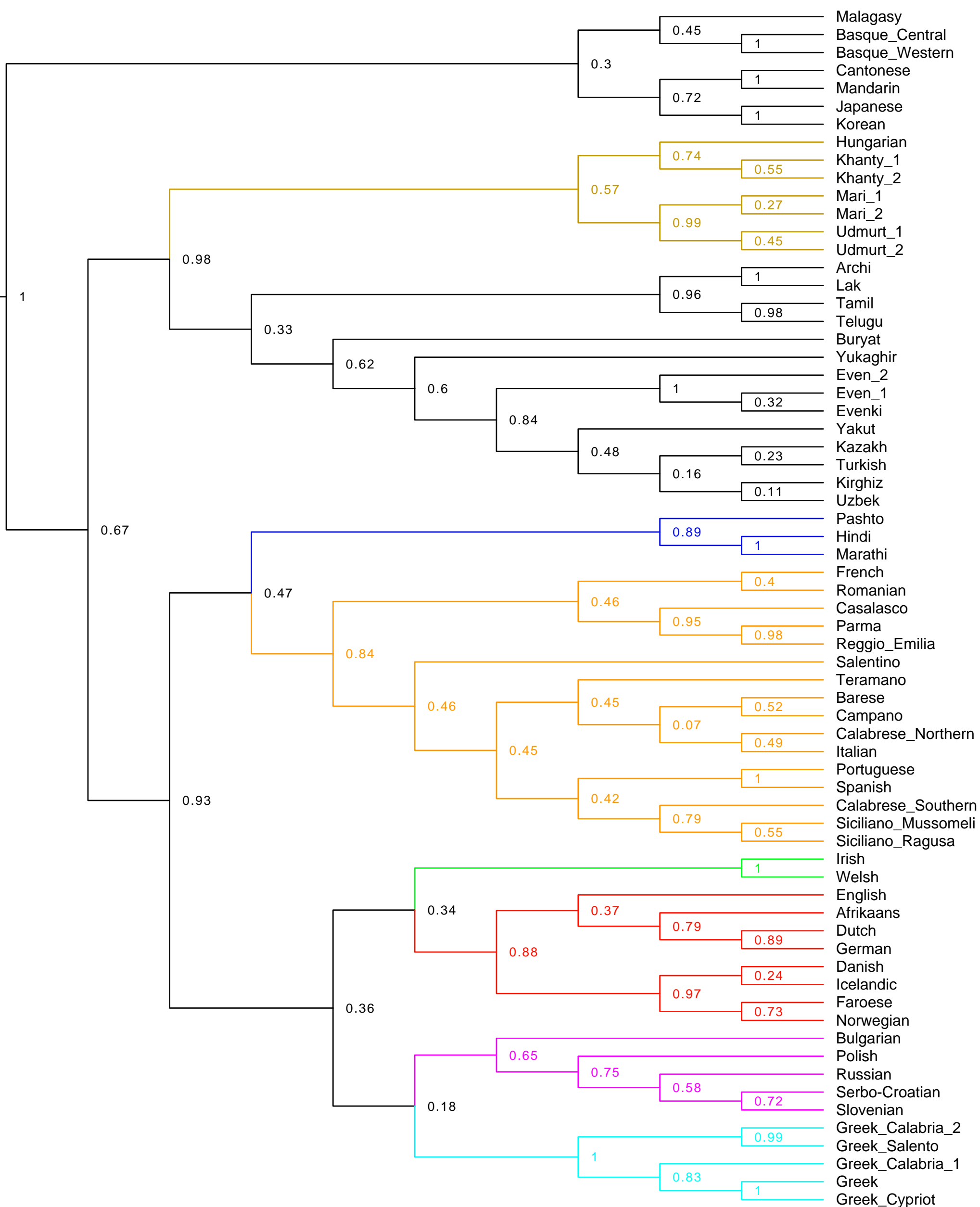

Supplement: Supplementary file 10 [file Image_9.pdf]

0.01

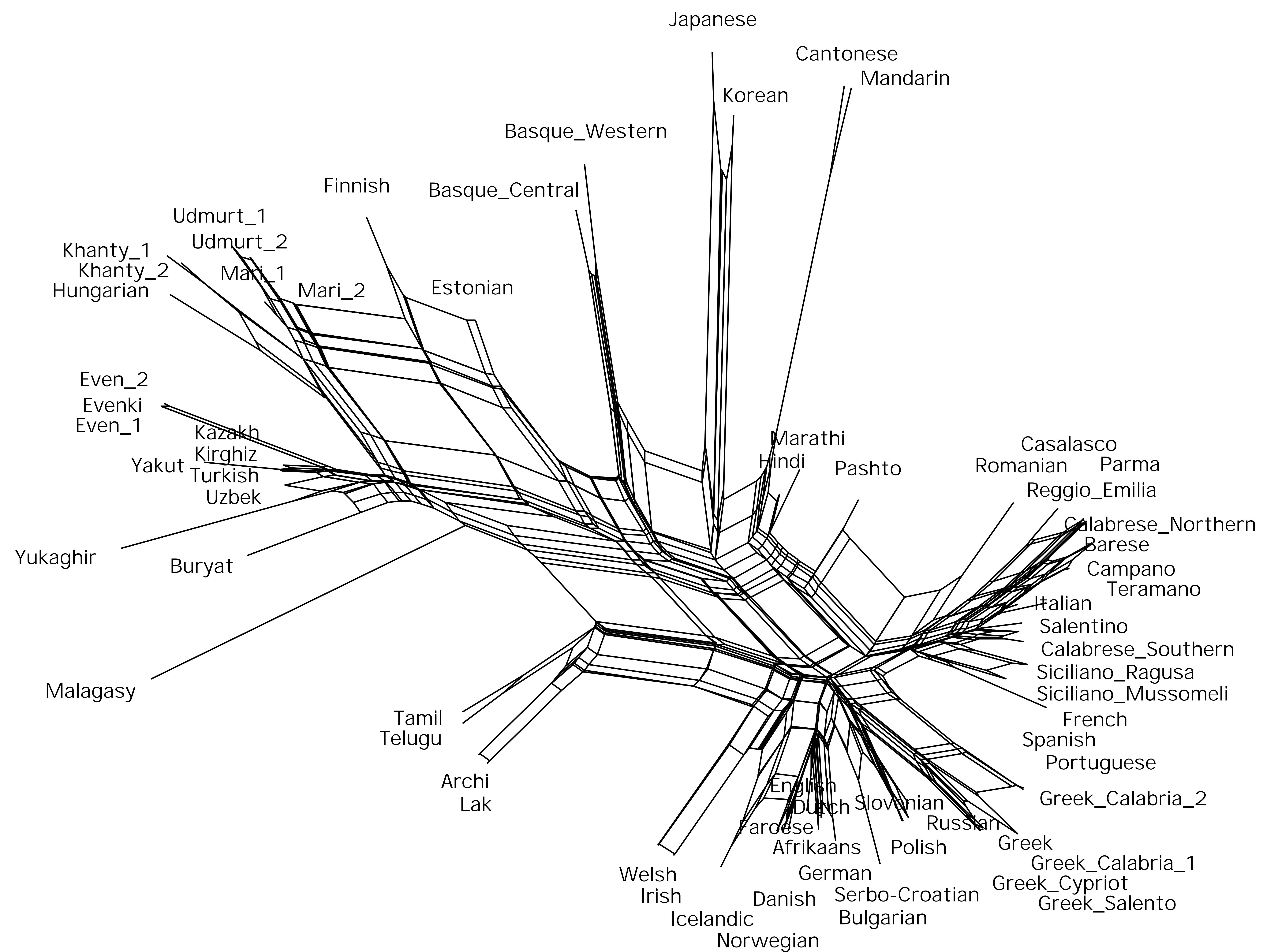

Supplement: Supplementary file 11 [file Image_10.pdf]

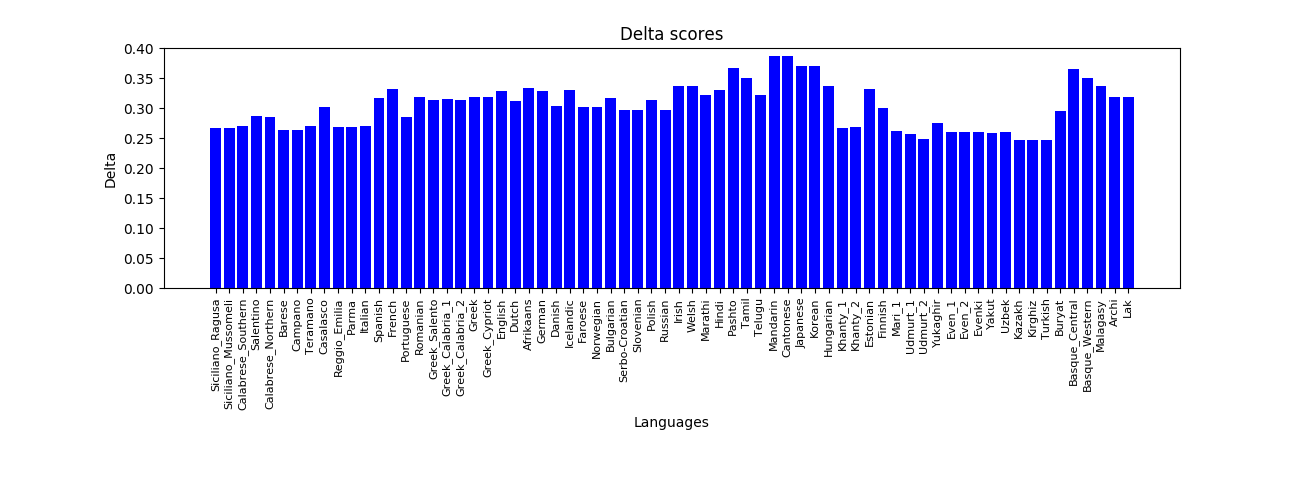

Supplement: Supplementary file 12 [file Image_11.png]

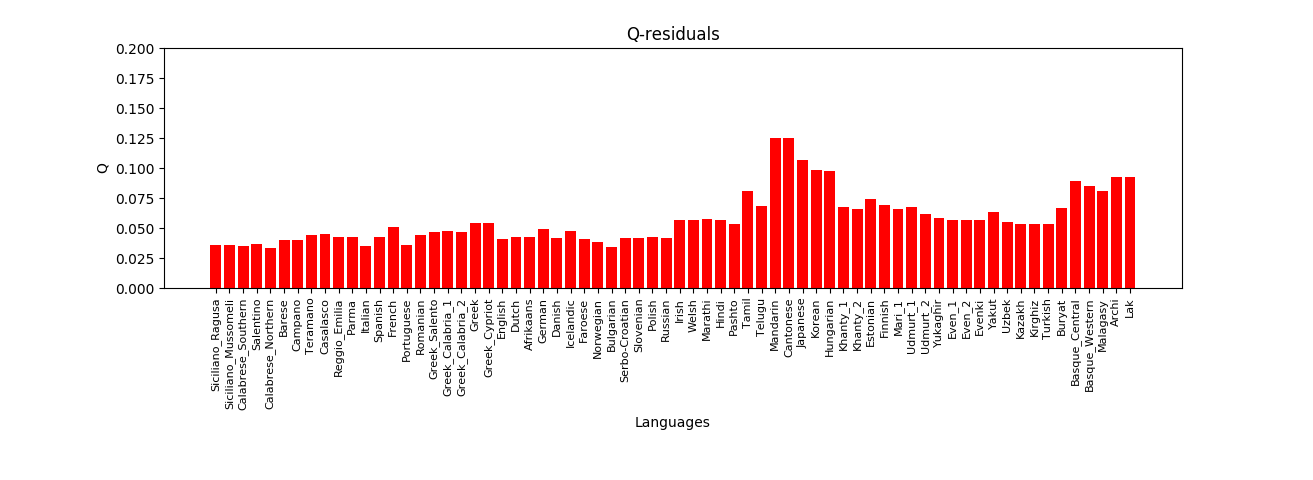

Supplement: Supplementary file 13 [file Image_12.png]

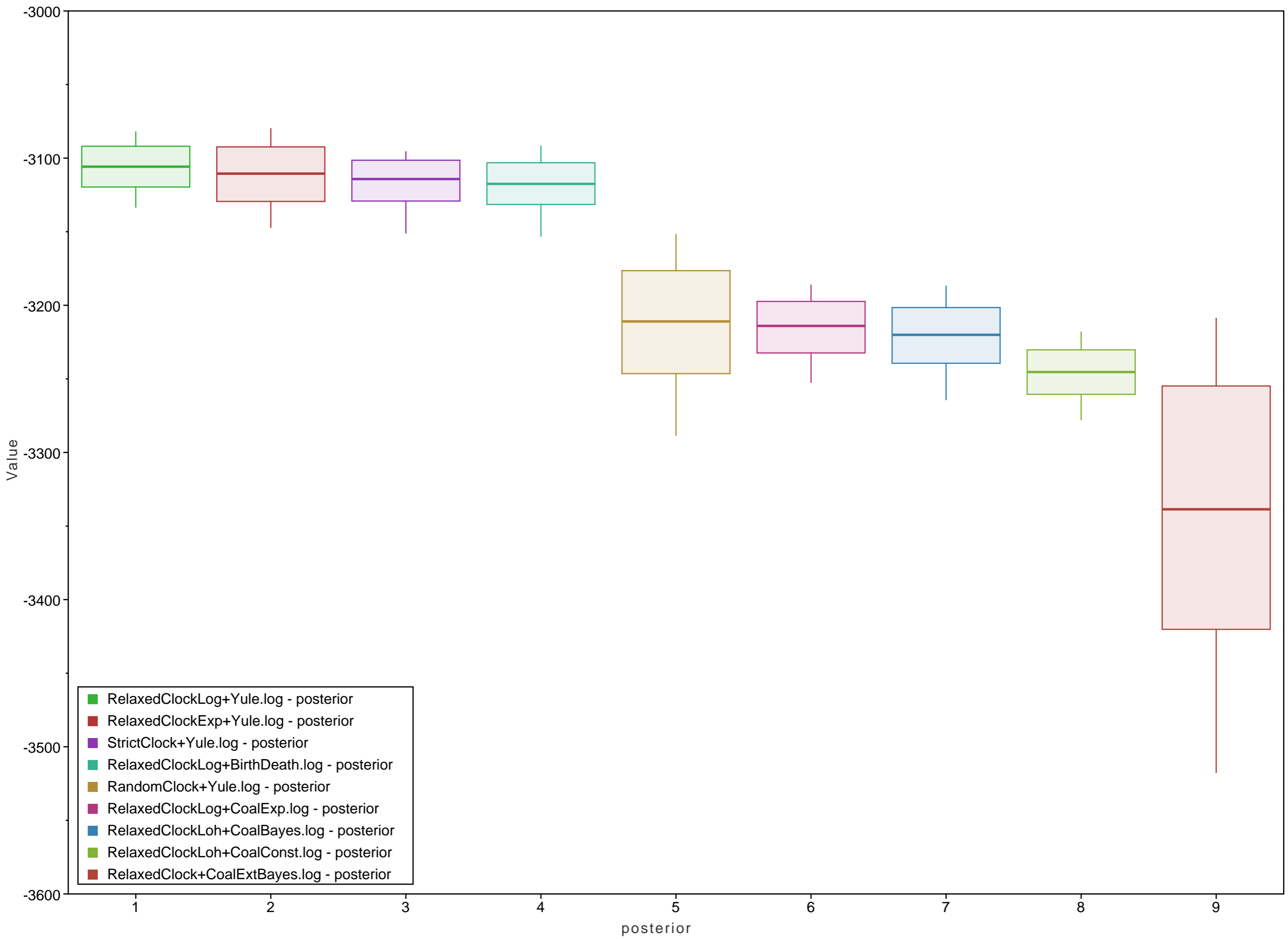

Supplement: Supplementary file 14 [file Image_13.pdf]

0.01

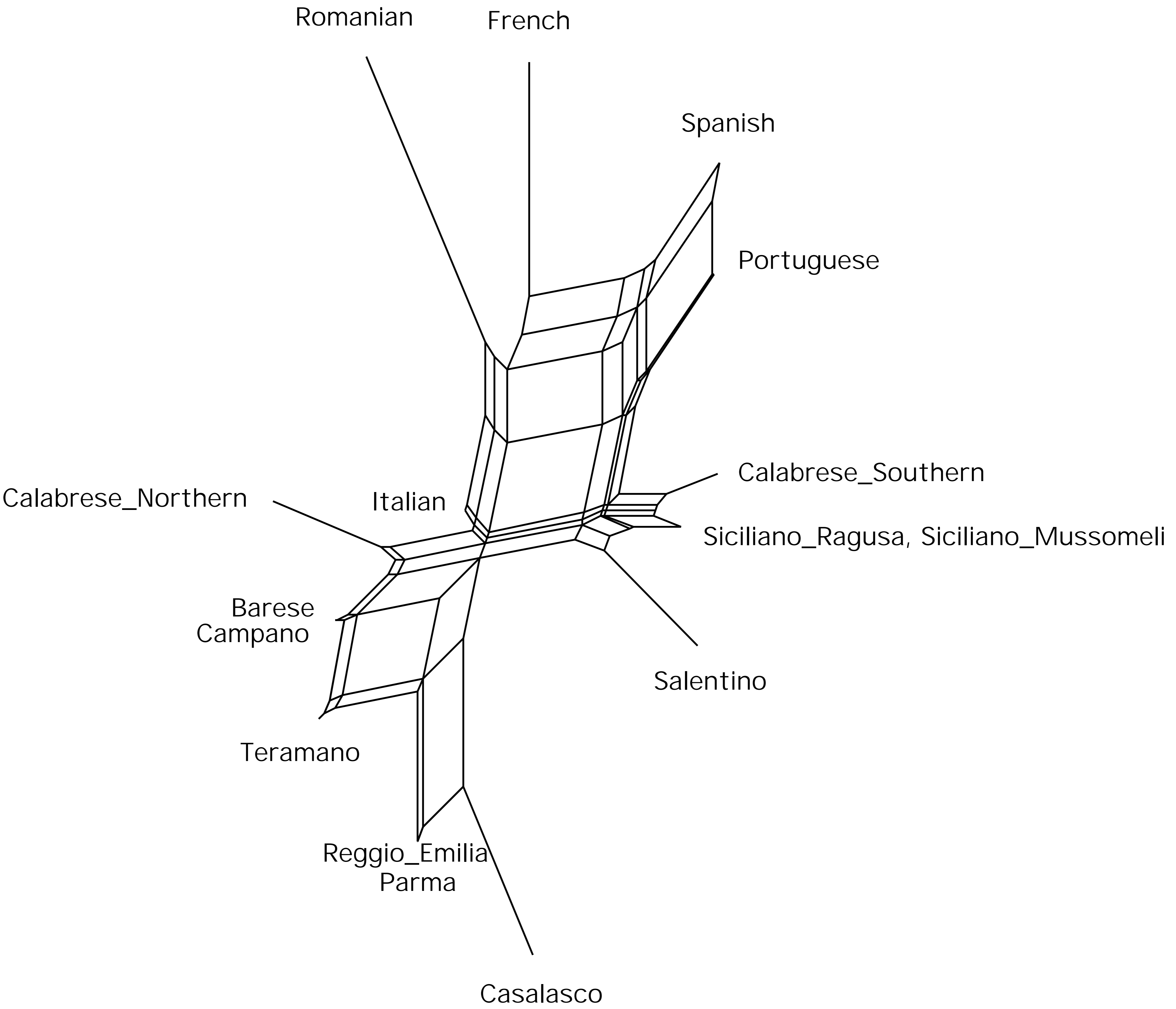

Supplement: Supplementary file 15 [file Image_14.pdf]

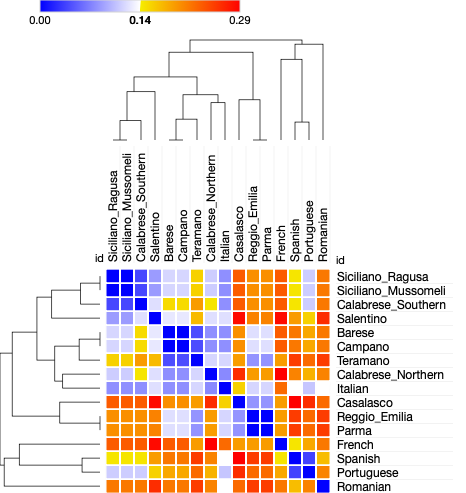

Supplement: Supplementary file 16 [file Image_15.png]
