# Supplementary material for: Formal Syntax and Deep History
Source: Front Psychol. 2020 Dec 18;11:488871. doi: 10.3389/fpsyg.2020.488871 (PMC7775603; doi:10.3389/fpsyg.2020.488871)
Supplement: Supplementary file 17 [file Presentation_1.pdf]

Formal syntax and deep history
Supplementary Material

Andrea Ceolin, Cristina Guardiano, Monica Alexandrina-Irimia, Giuseppe Longobardi*

*** Correspondence:**
Corresponding Author
giuseppe.longobardi@york.ac.uk

Keywords: phylogeny, formal syntax, parameters, language reconstruction, biolinguistics

1. Online Repository 1
2. Languages 1
3. The syntactic dataset 3
4. Possible languages 5
5. Heatmap - Syntactic Distances 5
6. PCoAs 7
7. Phylogenetic analysis - UPGMA 11
8. Phylogenetic analysis - Hamming distances 12
9. Phylogenetic analysis – BEAST 2 14
10. Network analysis - NeighborNet 18
11. Phonemic data - the Ruhlen Database 19
12. Ultralocality 20

1. Online Repository

The source code to replicate all the figures and the experiments presented in the paper and in the Supplementary Material is found in the following online repository (along with other relevant data and information): https://github.com/AndreaCeolin/FormalSyntax

2. Languages

The 69 languages of the dataset, along with their associated Glottolog (https://glottolog.org/glottolog/language) and ISO 639-3 codes, the family and subfamily they belong to, their location and geographic coordinates, are listed in **Supplementary Table 1**.

Language	Label	Glottocode	Iso 639-3	Top-level family	Family	Location	Latitude	Longitude
Afrikaans	Afk	afri1274	afr	IE	Germanic	Cape Town	-33.91	18.42
Archi	Arc	arch1244	aqc	NE Caucasian	-	Machačkala	42.01	47.26
Barese	BA	pugl1238	nap	IE	Romance	Bari	41.11	16.87
Basque_Central	cB	guip1235	eus	Basque	Guipuzcoan	Vitoria-Gasteiz	42.85	-2.68
Basque_Western	wB	bisc1236	eus	Basque	Biskayan	Bilbao	43.26	-2.93
Bulgarian	Blg	bulg1262	bul	IE	Slavic	Sofia	42.7	23.32
Buryat	Bur	buri1258	bua	Mongolic	E Mongolic	Ulan-Ude	51.82	107.61
Calabrese_Northern	NCA	sout3126	nap	IE	Romance	Verbicaro	39.75	15.19
Calabrese_Southern	SCA	sout2616	scn	IE	Romance	Reggio Calabria	38.11	15.66
Campano	Cam	napo1241	nap	IE	Romance	S.M. Capua Vetere	41.08	14.25
Cantonese	Can	cant1236	yue	Sino-Tibetan	Sinitic	Hong Kong	22.4	114.11
Casalasco	CR	west2342	egl	IE	Romance	Casalmaggiore	44.98	10.42
Danish	Da	dani1285	dan	IE	Germanic	Copenhagen	55.68	12.57
Dutch	Du	dutc1256	nld	IE	Germanic	Amsterdam	52.37	4.89
English	E	stan1293	eng	IE	Germanic	London	51.51	-0.13
Estonian	Est	esto1258	ekk	Uralic	Finno-Ugric	Tallinn	59.44	24.75
Even_1	Ev1	even1260	eve	Tungusic	N Tungusic	Kustur	67.79	130.4
Even_2	Ev2	even1260	eve	Tungusic	N Tungusic	Sebyan-Kyuyol	65.29	130.01
Evenki	Ek	even1259	evn	Tungusic	NW Tungusic	Bomnak	54.71	128.86
Faroese	FO	faro1244	fao	IE	Germanic	Tórshavn	62.01	-6.77
Finnish	Fin	finn1318	fin	Uralic	Finno-Ugric	Helsinki	60.17	24.94
French	Fr	stan1290	fra	IE	Romance	Paris	48.86	2.35
German	D	stan1295	deu	IE	Germanic	Berlin	52.52	13.4
Greek	Grk	mode1248	ell	IE	Greek	Athens	37.98	23.73
Greek_Calabria_1	CG1	aspr1238	ell	IE	Greek	Bova	37.99	15.92
Greek_Calabria_2	CG2	aspr1238	ell	IE	Greek	Bova Marina	37.93	15.55
Greek_Cypriot	CyG	cypr1249	ell	IE	Greek	Larnaca	34.09	33.62
Greek_Salento	SaG	apul1237	ell	IE	Greek	Calimera	40.24	18.27
Hindi	Hi	hind1269	hin	IE	Indo-Aryan	New Delhi	28.61	77.21
Hungarian	Hu	hung1274	hun	Uralic	Finno-Ugric	Budapest	47.5	19.04

**Icelandic**	Ice	icel1247	isl	IE	Germanic	Reykjavik	64.14	-21.94
**Irish**	Ir	iris1253	gle	IE	Celtic	Dublin	53.35	-6.26
**Italian**	It	ital1282	ita	IE	Romance	Rome	41.9	12.5
**Japanese**	Jap	nucl1643	jpn	Japonic	-	Tokyo	35.69	139.69
**Kazakh**	Kaz	kaza1248	kaz	Turkic	Kipchak	Almaty	43.22	76.85
**Khanty_1***	Kh1	khan1279	kca	Uralic	Finno-Ugric	Kazym	63.7	67.24
**Khanty_2***	Kh2	khan1279	kca	Uralic	Finno-Ugric	Kazym	63.7	67.24
**Korean**	Kor	kore1280	kor	Koreanic	-	Seul	37.57	126.98
**Kirghiz**	Kyr	kirg1245	kir	Turkic	Kipchak	Bishkek	42.87	74.57
**Lak**	Lak	lakk1252	lbe	NE Caucasian	-	Kumukh	42.54	47.89
**Malagasy**	Mal	plat1254	plt	Austronesian	Malayo-Polinesian	Antananarivo	18.88	47.51
**Mandarin**	Man	mand1415	cmn	Sino-Tibetan	Sinitic	Beijing	39.9	116.41
**Marathi**	Ma	mara1378	mar	IE	Indo-Aryan	Mumbai	19.08	72.88
**Mari_1***	mM1	mari1278	chm	Uralic	Mari	Shap	56.44	47.96
**Mari_2***	mM2	mari1278	chm	Uralic	Mari	Shap	56.44	47.96
**Norwegian**	Nor	norw1258	nor	IE	Germanic	Oslo	59.91	10.75
**Parma**	PR	cent1959	egl	IE	Romance	Parma	44.8	10.32
**Pashto**	Pas	pash1269	pus	IE	Iranian	Khyber Pass	34.09	71.16
**Polish**	Po	poli1260	pol	IE	Slavic	Warsaw	52.23	21.01
**Portuguese**	Ptg	port1283	por	IE	Romance	Lisbon	38.72	-9.1
**Reggio_Emilia**	RE	cent1959	egl	IE	Romance	Reggio Emilia	44.7	10.63
**Romanian**	Rm	roma1327	ron	IE	Romance	Bucharest	44.43	26.1
**Russian**	Rus	russ1263	rus	IE	Slavic	Moscow	55.76	37.62
**Salentino**	Sal	pugl1238	scn	IE	Romance	Cellino San Marco	40.47	17.96
**Serbo-Croatian**	SC	sout1528	hbs	IE	Slavic	Zagreb	45.82	15.98
**Siciliano_Mussomeli**	MsS	cent1963	scn	IE	Romance	Mussomeli	37.57	13.75
**Siciliano_Ragusa**	RGS	sout2617	scn	IE	Romance	Ragusa	36.92	14.72
**Slovenian**	Slo	slov1268	slv	IE	Slavic	Ljubljana	46.06	14.51
**Spanish**	Sp	stan1288	spa	IE	Romance	Madrid	40.42	-3.7
**Tamil**	Ta	tami1289	tam	Dravidian	-	Madras	13.08	80.27
**Telugu**	Te	telu1262	tel	Dravidian	-	Hyderabad	17.39	78.49
**Teramano**	Ter	neap1235	nap	IE	Romance	Teramo	42.66	13.7
**Turkish**	Tur	nucl1301	tur	Turkic	Oghuz	Ankara	39.93	32.86
**Udmurt_1***	Ud1	udmu1245	udm	Uralic	Permian	Chur	57.07	53.03
**Udmurt_2***	Ud2	udmu1245	udm	Uralic	Permian	Chur	57.07	53.03
**Uzbek**	Uz	uzbe1247	uzb	Turkic	Turkestan Turkic	Tashkent	41.3	69.24
**Welsh**	Wel	wels1247	cym	IE	Celtic	Cardiff	51.48	-3.18
**Yakut**	Ya	yaku1245	sah	Turkic	N Siberian Turkic	Jakutsk	62.04	129.68
**Yukaghir**	Yu	yuka1259	yux	Yukaghir	Kolmic (S Yukaghir)	Kolyma	65.5	151.09

Supplementary Table 1. The languages of the dataset. For the languages marked with '*' we encoded two diastratic varieties.

3. The syntactic dataset

Supplementary Figure 1 contains the 94 binary nominal parameters used for the experiments presented in the paper, set in 69 languages spanning across up to 13 traditionally irreducible Eurasian families.

The table should be read as follows:

1st column: progressive number of the parameters (p1, p2, p3, …)

2nd column: acronym of the parameter

3rd column: name of the parameter

4th column: implicational constraints specifying the conditions for setting the parameter

A detailed list of questions used to determine the state of the parameters and instructions to map them to **Supplementary Figure 1** can be found in:

Crisma, P., C. Guardiano and G. Longobardi (2020), Syntactic parameters and language learnability. *Studi e Saggi Linguistici* 58, 99-130. doi: 10.4454/ssl.v58i2.265

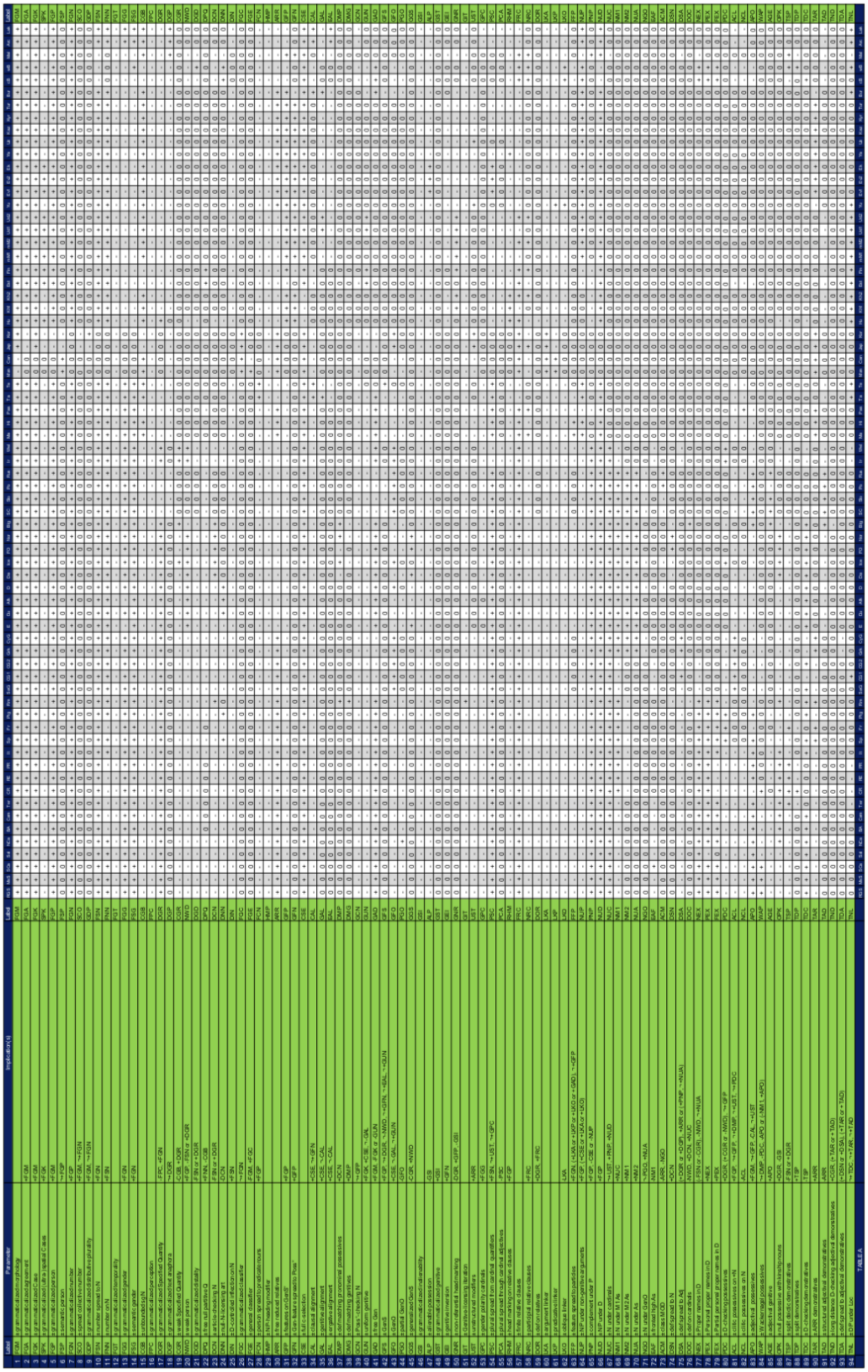

Supplementary Figure 1. 94 binary nominal parameters set in 69 languages.

4. Possible languages

We calculated the number of possible languages generated from the first 30 parameters of Table A using an algorithm first presented in Bortolussi et al. (2011). This is a breadth-first search algorithm that keeps track of the number of 'possible strings', namely, those strings which do not violate the implicational rules. The algorithm takes as input two strings of length=1, one with the value "+" and one with the value "-".Then, from each string it creates three different strings of length=2, adding one of the three possible values ("+", "-" and "0"), so that at the first iteration we have "+/+", "+/-", "+/0" and "-/+", "-/-", and "-/0", for a total of six possible strings. Before the next iteration, only the strings which are compatible with the implicational structures are kept, while the others are discarded. The procedure is then repeated, so that at each iteration only the strings which are compatible are kept.
We limited the analysis to the first 30 parameters because the algorithm has exponential complexity, and therefore, as every subset of strings needs to be triplicated at each iteration, the algorithm will take much more time to process every string at each following iteration (see the online repository for the Python script that we used). Through the algorithm, we calculated that the first 30 parameters used here generate only 152,448 possible languages (~2^{17}) instead of 2^{30}. These figures suggest that calculations of the probability of relatedness based on grammatical structure but neglecting the pervasive effect of such predictable information could be seriously undermined. We expect the rate of possible languages to increase at an even lower rate when more parameters are added to the search space, because they will be potentially constrained by higher numbers of previous parameters.

5. Heatmap - Syntactic Distances

Instructions to visualize the heatmap in **Figure 1** in the text.
1. Go to the page https://software.broadinstitute.org/morpheus/
2. Upload to the page the file *jaccard_distances.txt* from the GitHub repository (link: https://github.com/AndreaCeolin/FormalSyntax/blob/master/jaccard_distances.txt) and click the "OK" button to visualize the heatmap
3. In the "Tools" menu, select the option "Hierarchical clustering", and then the following options:
   a. Metric > Matrix values (from a precomputed distance matrix)
   b. Linkage method > average
   c. Cluster > Rows and columns

   Click the "OK" button.
4. To visualize the same color distribution as **Figure 1**, follow the instructions below:
   a. In the "View" menu, select "Options"
   b. In the "Color Scheme" window:
      i. Uncheck the "Relative color scheme" choice
      ii. "Maximum" > 0.857
      iii. "Add color stop"
      iv. "Selected color" > yellow
      v. "Selected value" > 0.430

Supplementary Table 2 lists the maximal clusters of cells in **Figure 1** which do not contain any yellow/red cell. The symbol δ refers to the Jaccard distance between two languages, the symbol μ

to the average distance among the languages belonging to a given aggregation/cluster, obtained as the mean of all the pairwise distances between the languages of that aggregation.
Supplementary Table 3 lists the subgroups which can be identified within each of the cluster in **Supplementary Table 2**, along with the distance range and mean within each subfamily.

**Maximal cluster**	**δ (range)**	**μ**
1. Indo-European	From 0 to 0.42	0.26
2. Dravidian+NE Caucasian	From 0.10 to 0.25	0.17
3A. Uralic	From 0.05 to 0.24	0.16
4A. Altaic+Yukaghir	From 0 to 0.32	0.16
3B. Balto-Finnic	0.13	0.13
4B. [rest of]Uralic+Altaic+Yukaghir	From 0 to 0.42	0.23
5. Basque	0.17	0.17
6. Sinitic	0.10	0.10
7. Korean-Japanese	0.17	0.17

Supplementary Table 2. Clusters of cells which do not contain any yellow/red cell in **Figure 1**.

**Subfamily**	**δ (range)**	**μ**
Romance	From 0 to 0.29	0.16
Greek	From 0 to 0.17	0.11
Germanic	From 0.04 to 0.19	0.12
Slavic	From 0 to 0.17	0.08
Indo-Iranian	From 0.05 to 0.24	0.16
Dravidian	0.10	0.10
NE Caucasian	0	0
Balto-Finnic	0.13	0.13
Ugric	From 0.07 to 0.19	0.14
Permic-Volgaic	From 0.05 to 0.11	0.07
Tungusic	0	0
Turkic	From 0 to 0.12	0.05

Supplementary Table 3. Distances and means within the subfamilies identifiable in **Figure 1**.

Other observations:

a. Only one pair formed by a member of **Cluster 1** (IE) and a language outside of it has δ<0.26 (i.e. lower than the μ of the cluster), i.e. Ma-Ta (0.25); two pairs have δ=0.26 (Ma-Te and Hi-Te). Overall, there are 185 white/blue cells (δ < 0.429) involving a member of **Cluster 1** and a language outside of it. Most such pairs contain one Indo-Iranian language and one Dravidian, NE Caucasian, Uralic or Altaic language.

b. All the members of **Cluster 2** display many similarities with other languages of the sample: overall, 93 pairs involving either of the two Dravidian languages and one Indo-European, Uralic or Altaic language, and 90 pairs involving either of the two NE Caucasian languages and one Indo-European, Uralic or Altaic language are either white or light blue, with δ ranging from 0.25 to 0.42.

c. Almost all the languages belonging to **Cluster 3A** display similarities with many other languages outside of it (124 blue/white cells), notably Indo-Iranian, Dravidian, NE Caucasian, Altaic, Yukaghir and (to a smaller extent) Malagasy and Basque, with δ ranging from 0.19 to 0.42.

d. As far as **Cluster 4A** is concerned, there are 163 blue/white cells involving one of its members with a language outside of the cluster, and most of them involve Indo-Iranian, Dravidian, NE Caucasian, Uralic and, marginally, Malagasy and other IE languages.
Buryat and Yukaghir are the outliers of the cluster: yet, no aggregation of blue/white cells containing either of the two languages displays a μ/δ smaller than those they hold with the rest of Cluster 4A (see **Supplementary Table 4**).

e. The languages sharing non-yellow/red cells with Malagasy (an isolate in the Heatmap) are: Mari_2 (δ=0.39), Udmurt_2 (δ=0.42), Uzbek, Kazakh, Kirghiz, Turkish (δ=0.40).

f. The languages sharing non-yellow/red cells with **Cluster 5** (Basque) are: Mari_2 (δ[Basque_Western]=0.41, δ[Basque_Central]=0.40), Marathi (δ[Basque_Western]=0.39) and Pashto (δ[Basque_Western]=0.41).

g. Sinitic languages (**Cluster 6**) do not share any white/blue cell with other languages of the sample, with the exception of Hindi (δ = 0.38).

h. There are two languages which share non-yellow/red cells with **Cluster 7**, i.e., Greek and Cypriot Greek (δ = 0.42 with Japanese, and δ = 0.36 with Korean).

**Language**	**Cluster (Language)**	**μ/δ**
Buryat	Cluster 4A (Tungusic, Turkic, Yukaghir)	0.25
	Cluster 4B (Ugric, Permic-Volgaic, Altaic, Yukaghir)	0.27
	Cluster 2 (Dravidian+NE Caucasian)	0.34
	Indo-Aryan [Cluster 1]	0.35
Yukaghir	Cluster 4A (Tungusic, Turkic, Buryat)	0.25
	Cluster 4B (Ugric, Permic-Volgaic, Altaic)	0.28
	Cluster 2 (Dravidian+NE Caucasian)	0.39
	Indo-Aryan [Cluster 1]	0.40

Supplementary Table 4. Relations between Buryat/Yukaghir and closest languages.

6. PCoAs

The PCoAs have been produced using the software PAST (https://www.nhm.uio.no/english/research/infrastructure/past/). After the distance matrix is loaded, the following option should be selected: *Multivariate -> Ordination -> PCoA.*

In the scatter plot, the attribute *Row Labels* must be selected to display the name of the languages.
The PCoA in **Supplementary Figure 2** was obtained from the parametric Jaccard distances between the 30 non-Indo-European languages of our sample.
In **Supplementary Figure 2**, the first coordinate, which accounts for about 58% of the variance, separates Uralic, Dravidian, NE Caucasian, Altaic, and Yukaghir (left area) from the others.

a. Left area: the second coordinate (accounting for 17% of the variance) separates Altaic (with Buryat falling precisely on the horizontal axis) and Yukaghir (bottom quadrant) from the rest. In the top quadrant, Uralic, Dravidian and NE Caucasian are not clearly separated: this reflects the high amount of similarities among these languages observed in the Heatmap.

b. Right quadrant: the second coordinate separates the languages of the Far-East (bottom quadrant) from the rest. Japanese and Korean, which appear very close to one another in the Heatmap, in this representation are quite separated.

As it appears in the graph, distances, especially in the left quadrant, are quite compressed: hence, the internal distribution of the pairs does not emerge clearly. In order to observe it in more detail, we visualized the two groups identified by the first coordinate as two separate graphs, shown in **Supplementary Figure 3** and **Supplementary Figure 4.**
The distribution of the pairs in **Supplementary Figure 3** further emphasizes the neat separation between Sinitic and Japanese-Korean.
In **Supplementary Figure 4**:

a. Dravidian and NE Caucasian are a separate cloud (top right quadrant).
b. The top left quadrant shows two major clouds:
   i. all Altaic languages but Buryat
   ii. Buryat (that expectedly appears as an outlier of the group) and Yukaghir (that, again, is attracted by the Altaic group)
c. Uralic forms a relatively compact cloud in the bottom area of the graph, with Estonian and Finnish in an outlying position, as seen in the Heatmap.

Finally, **Supplementary Figure 5** contains the 39 IE languages of our sample. Their distribution partitions the known subfamilies with a discrete resolution and without historical errors. The first coordinate, which accounts for 46% of the variance, separates Romance from the other subfamilies. In the left area, the horizontal axis (which accounts for 18% of the variance) identifies:

a. Germanic and Slavic, which form two separate clouds in the bottom-left quadrant
b. Celtic, Greek and Indo-Iranian (more scattered)

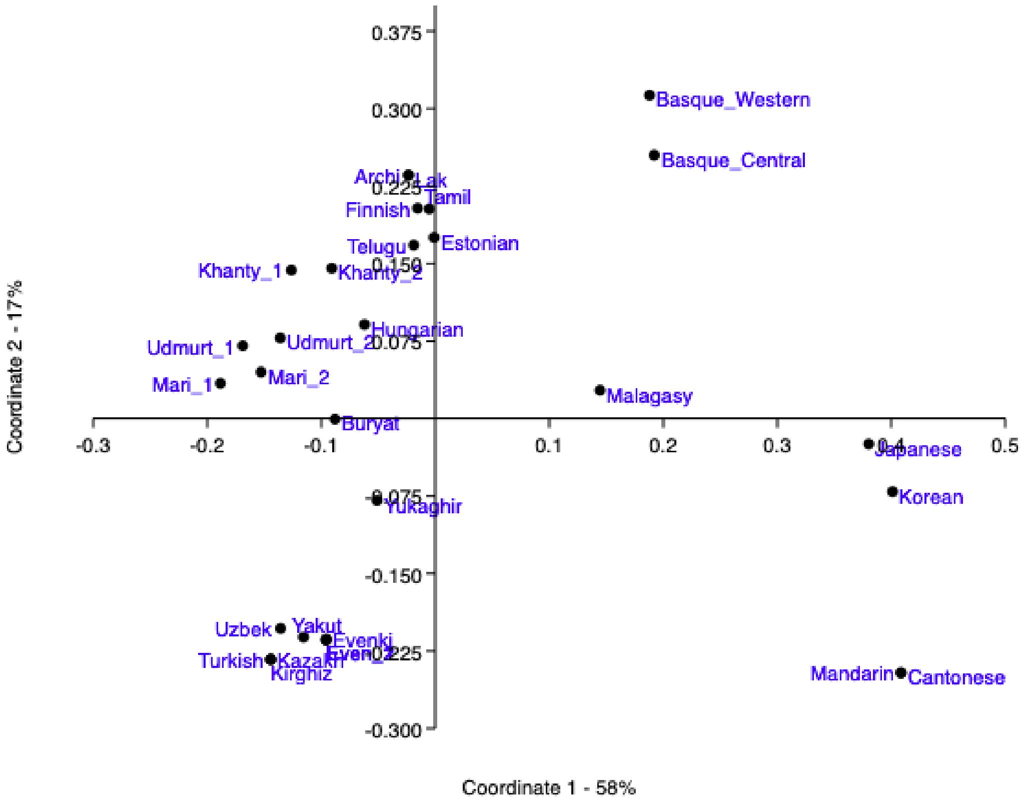

Supplementary Figure 2. PCoA of the 30 non-Indo-European languages.

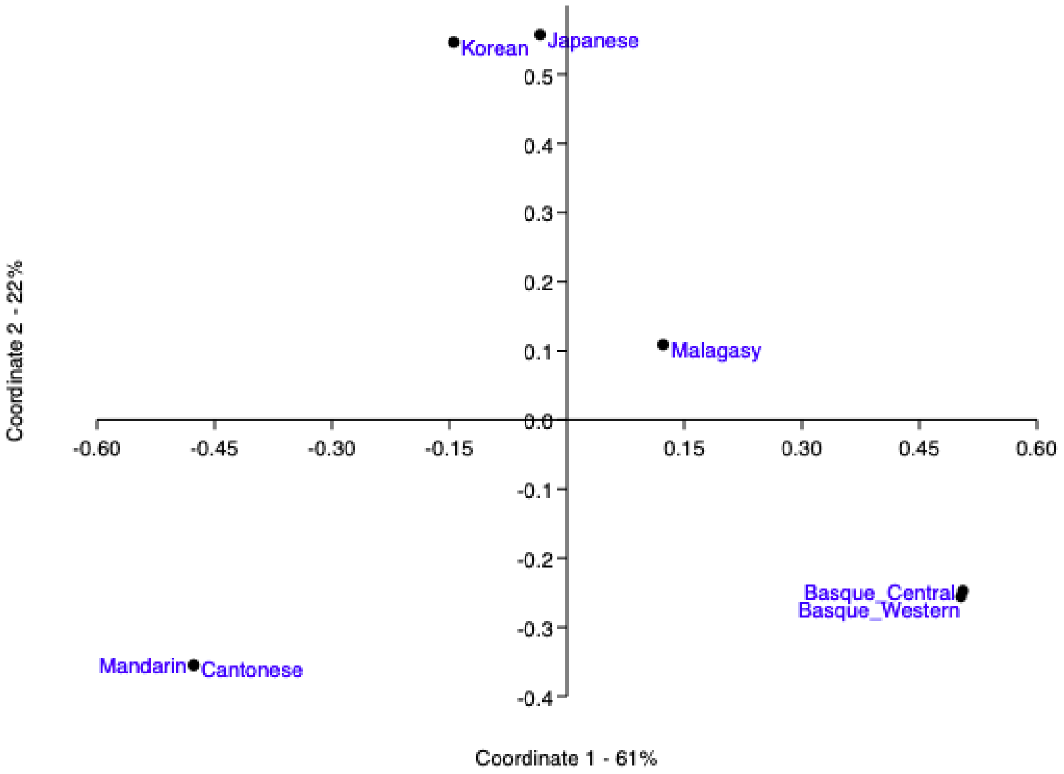

Supplementary Figure 3. PCoA of 7 non-Indo-European languages.

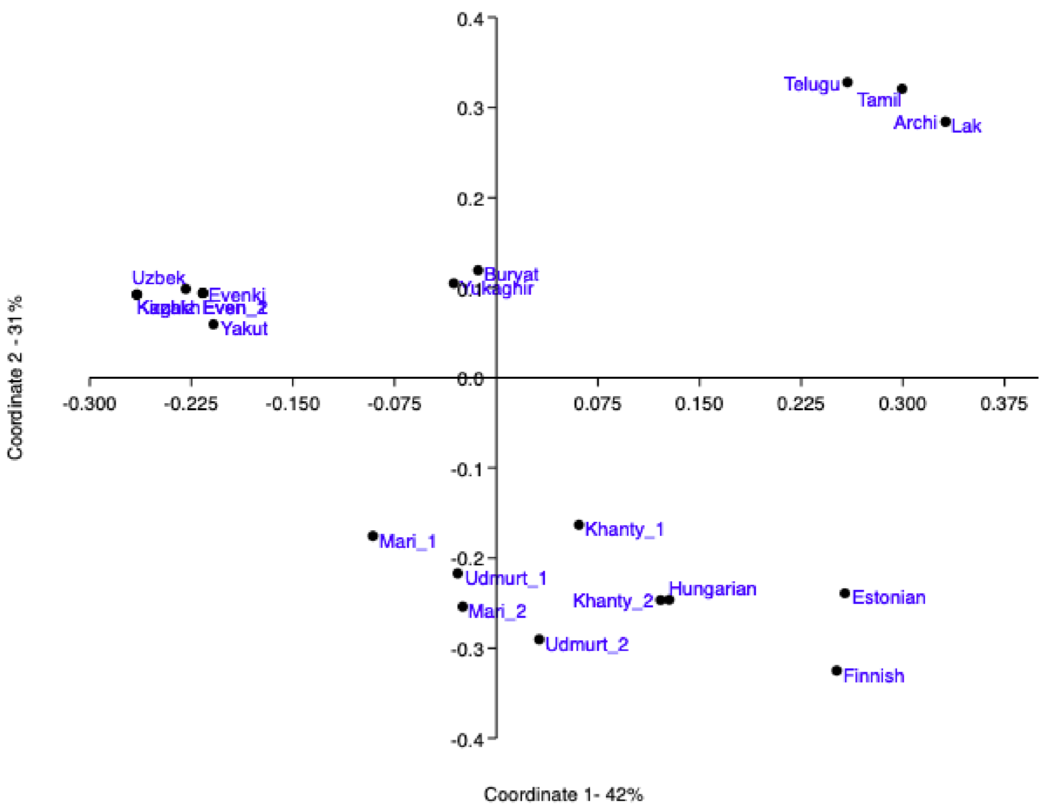

Supplementary Figure 4 - PCoA of 18 non-Indo-European languages.

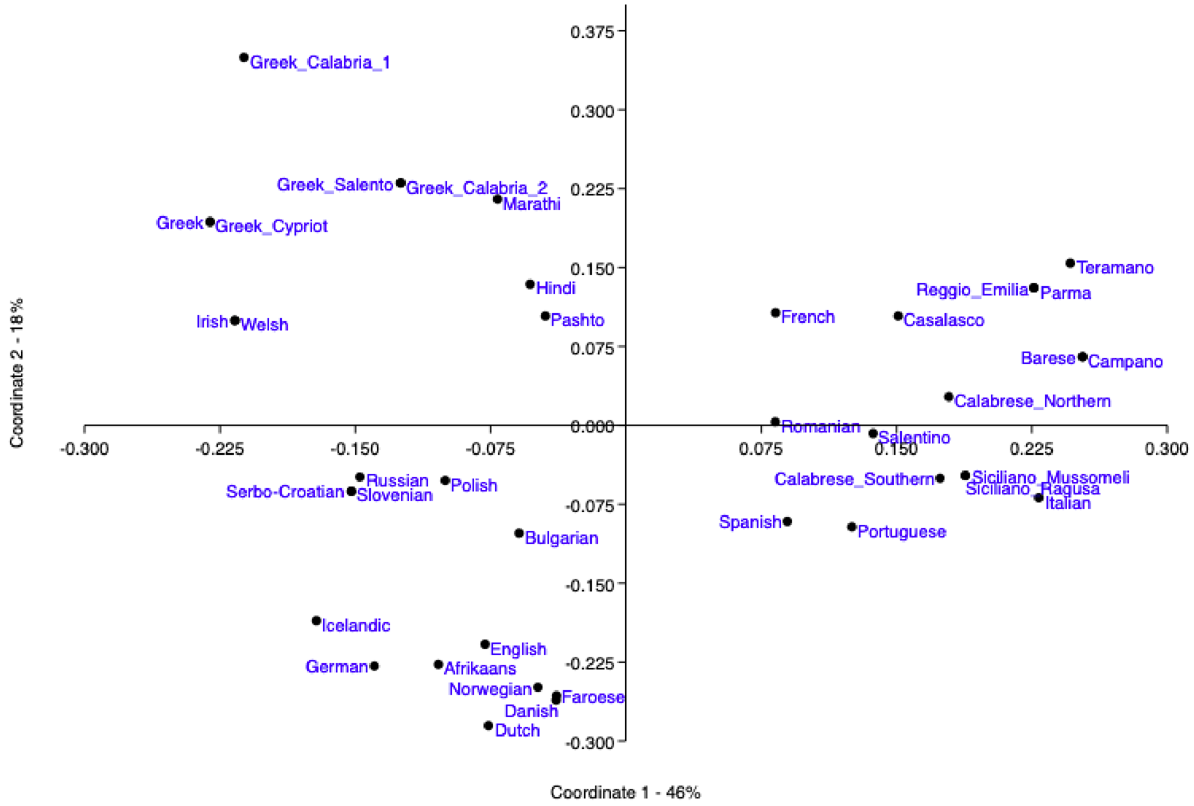

Supplementary Figure 5 - PCoA of the 39 Indo-European languages.

7. Phylogenetic analysis - UPGMA

The UPGMA tree (**Figure 3** in the main text) has been generated using a modified bootstrapping procedure.

The bootstrapping technique resamples the whole dataset by selecting each character with equal probability and recreating a matrix of the same length. The content of the new matrix is different from the original matrix, because some characters might be absent and some others might be present multiple times as a consequence of the sampling procedure. This allows one to estimate the robustness of the dataset by repeating the same analysis on different samples of the dataset.

Since the Jaccard distance between two languages excludes all parameters that are set to '0' in either one of them, a standard bootstrapping procedure runs the risk of making a pair of languages not comparable, because in some replicas the number of identities plus differences can reduce to zero, and then yield a zero denominator for the Jaccard formula. For this reason, we decided to adopt a moderated bootstrap procedure, by creating 1000 datasets in which only six parameters are resampled. Since the minimum number of comparable parameters between any two languages in the dataset is seven, a resampling of six parameters will assure that the two strings are comparable by means of the Jaccard distance.

The UPGMA tree presented in the text is a consensus tree resulting from applying UPGMA to the 1000 replicas of the dataset.

The first two splits of **Figure 3** identify the following nodes:

a. The languages spoken in East Asia, with Japanese and Korean falling under one and the same node
b. Basque

A further split separates two major clusters, internally articulated as follows:

1. a. Malagasy
   b. Uralic, articulated into the following groups:
      - Balto-Finnic
      - Ugric
      - Volgaic-Permic, with a low bootstrapping score, which shows that the two subfamilies are often mixed when replicating the experiment
2. a. Altaic+Yukaghir, with the following internal articulation
      - Yukaghir is the outlier
      - Buryat
      - Tungusic
      - Turkic: Kazakh and Kirghiz are clustered together, followed in succession by Turkish, Yakut and Uzbek (NE Turkic). Note the low bootstrapping score of the Kazakh and Kirghiz node, which means that by replicating the experiment they might end up clustering with Turkish first.
   b. i. NE-Caucasian and Dravidian
      ii. Indo-European, articulated into the following major subfamilies:
         - Indo-Iranian. Pashto is the outlier. The two Indo-Aryan languages are together
         - Romance. Romanian is the outlier. The Ibero-Romance unit (Spanish and Portuguese) is recognized. The dialects of Italy, and Italian, are under the same node, with the following internal articulation: Northern Gallo-Italic dialects (Casalasco, Parma and Reggio_Emilia); Extreme-southern dialects (Salentino, Calabrese_Southern and Siciliano); Upper-southern dialects (Teramano, Barese, Campano and Calabrese_Northern) and Italian
         - Celtic
         - Greek. Greek clusters with Greek_Cypriot; Greek_Calabria_1 is the outlier of this group, reflecting its documented conservative nature (Guardiano et al. 2016, Guardiano and Stavrou 2014, 2019)
         - Slavic. Bulgarian occurs as the outlier. Polish and Russian fall together
         - Germanic. Three out of four traditional West-Germanic languages (Continental West Germanic) are under one and the same node (Afrikaans, Dutch and German). English falls within the North-Germanic cluster (Icelandic, Danish, Faroese, Norwegian)

8. Phylogenetic analysis - Hamming distances

We created a UPGMA tree (**Supplementary Figure 6**) from a matrix of Hamming distances, using the same procedure as for **Figure 3**. The tree retrieves most of the nodes observed in **Figure 3,** with three major differences:

a. Pashto and Romanian go together
b. the nodes containing the two Basque varieties, the two Sinitic languages and Japanese/Korean are not the outliers (they are closer to the Indo-European node)
c. West-Germanic and a North-Germanic node are identified

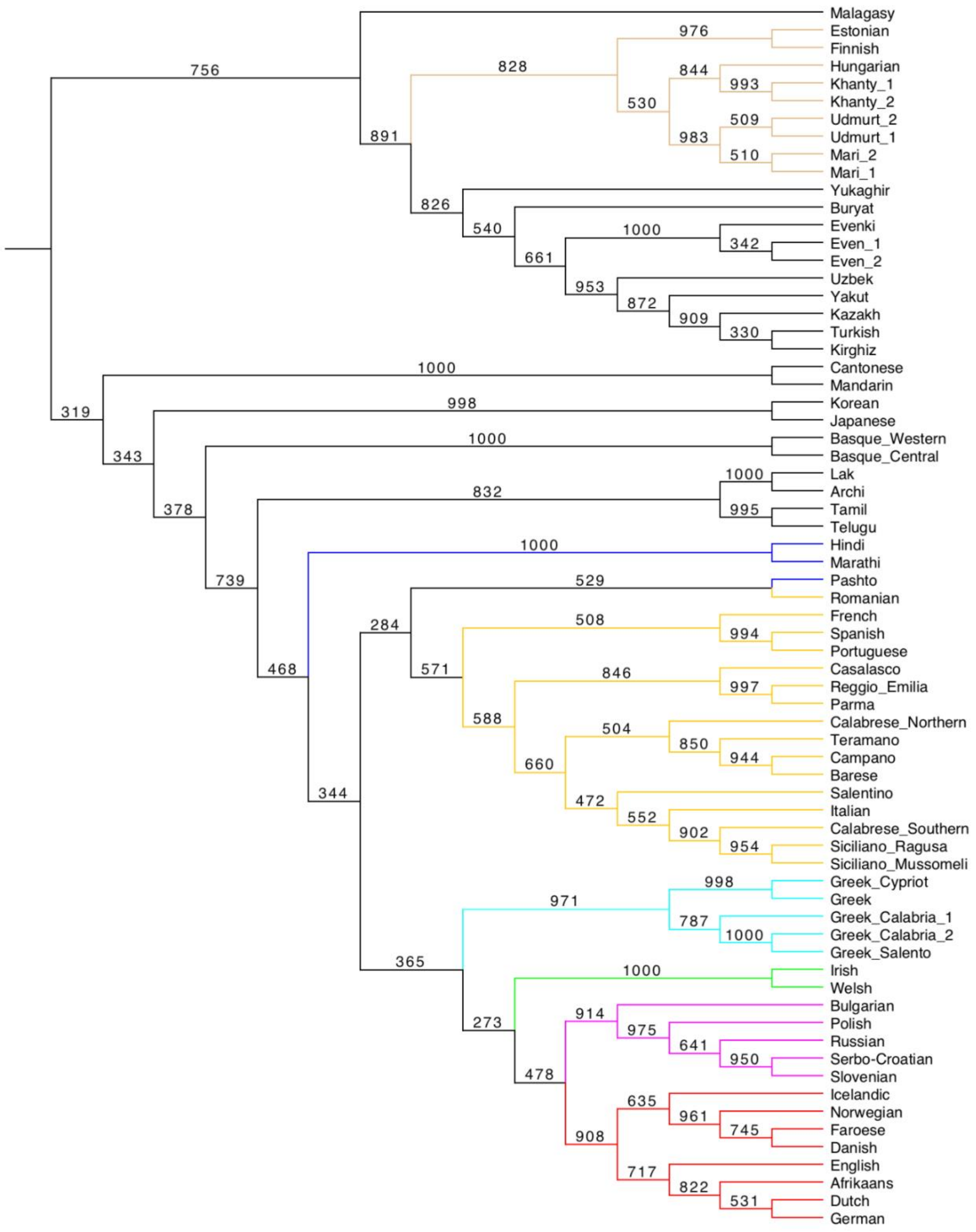

Supplementary Figure 6. UPGMA tree calculated using Hamming distances.

9. Phylogenetic analysis – BEAST 2

In order to determine the best model for the BEAST tree (Bouckaert et al. 2019), we used the software Tracer (https://beast.community/tracer) to compare the posterior likelihood of several models. The analysis is summarized in **Supplementary Figure 7.** The best model that we determined is a Gamma Site Model with Substitution Rate = 1, a Mutation Death Model with death p = 0.1, a Relaxed Clock (Logarithmic) with clock rate = 1, and a uniform Yule model for the birth rate. The Monte Carlo Markov Chain produced 10,000,000 trees, 25% of which were used for the burn-in and discarded for the purpose of the calculation of the consensus tree. The tree is a consensus tree of 7,500 different trees sampled through the 7,500,000 trees (with a sample stored every 1000 generated trees) produced by Monte Carlo sampling.

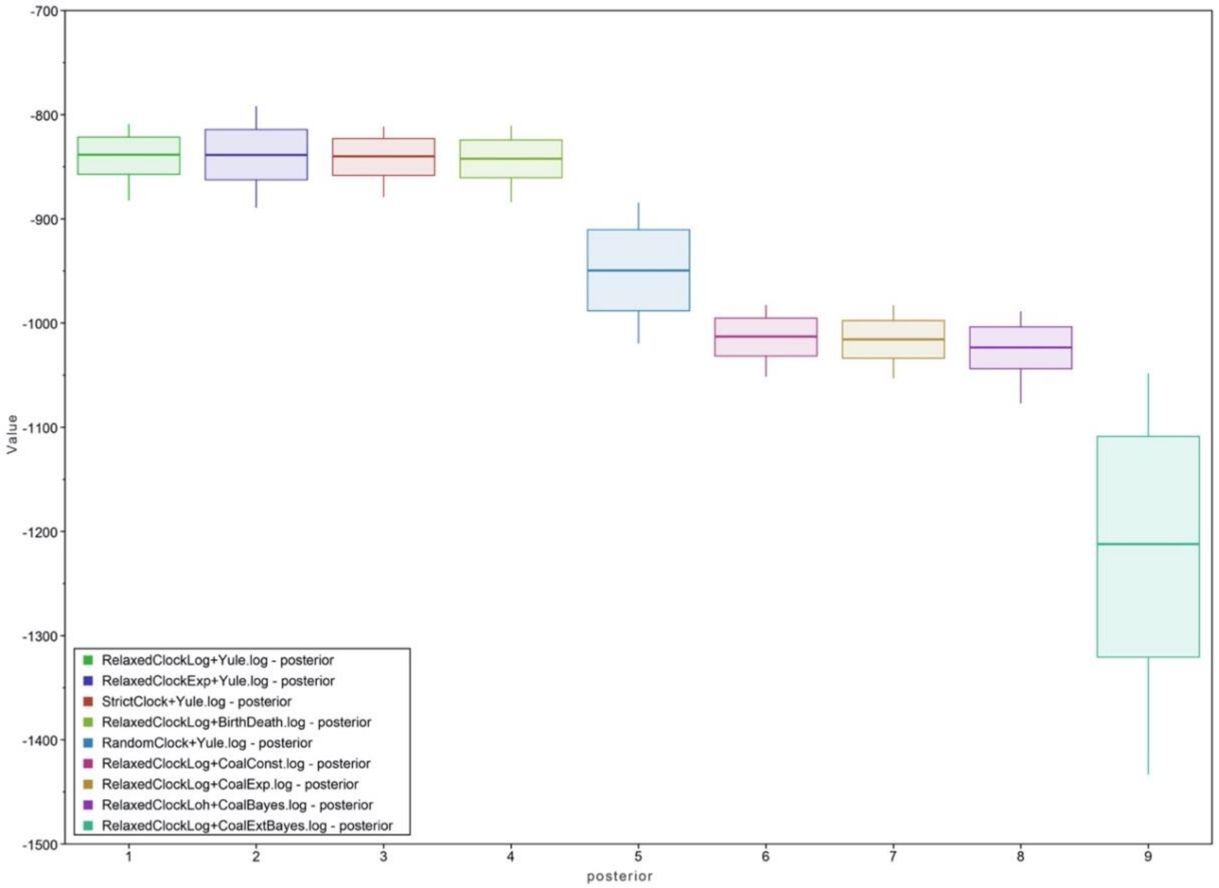

Supplementary Figure 7. Tracer analysis for different BEAST models used to generate a tree from the syntactic dataset.

The constrained BEAST tree (**Figure 4** in the text) identifies the following splits:

a. The languages spoken in East Asia, with Japanese and Korean falling under one and the same node
b. Malagasy and Basque
c. Uralic, articulated into the following groups:
   - Balto-Finnic (Estonian and Finnish)

- Ugric (Hungarian and Khanty)
- Volgaic-Permic (Mari and Udmurt), with a low posterior probability, which means that the two subfamilies can appear mixed in some replications of the experiments

d. A node that splits into the following:
   - Dravidian (Tamil and Telugu) + NE Caucasian (Archi and Lak)
   - Altaic+Yukaghir, with the following internal articulation:
     - Buryat
     - Yukaghir
     - Tungusic (Even, Evenki)
     - Turkic: Kazakh and Kirghiz are clustered together, followed in succession by Turkish, Yakut and Uzbek. All these nodes have low posterior probability, which means that the internal articulation of the family is not defined, and therefore is not stable across different replications

e. Indo-European, articulated into the following major subfamilies:
   - Indo-Iranian. Pashto is the outlier of the two Indo-Aryan languages (Hindi, Marathi)
   - Romance. Romanian is the outlier. French is the outlier of a node that also includes the Northern Gallo-Italic dialects (Casalasco, Parma and Reggio_Emilia). Salentino is the outlier of a node that has the following splits: Italian and Upper southern dialects of Italy (Calabrese_Northern, Teramano, Barese, Campano); Ibero-Romance and the Extreme southern dialects of Italy (Calabrese_Southern, Siciliano_Mussomeli, Siciliano_Ragusa), with the exception of Salentino)
   - Celtic (Irish and Welsh) + Greek (with the same subarticulation as in UPGMA)
   - Slavic (with the same subarticulation as in UPGMA)
   - Germanic, split into West- vs. North-Germanic (contrary to UPGMA, both nodes are correctly identified)

Supplementary Figure 8 displays an unconstrained tree generated using BEAST. Here, Finnish and Estonian do not cluster with the other Uralic languages, but are the outliers of a group containing Uralic, NE Caucasian and Dravidian, Turkic, Tungusic, Buryat and Yukaghir. In other replications, Balto-Finnic appears as an outlier of the Indo-European languages, or even inside this family.
A tree without Finnish and Estonian is displayed in **Supplementary Figure 9.**

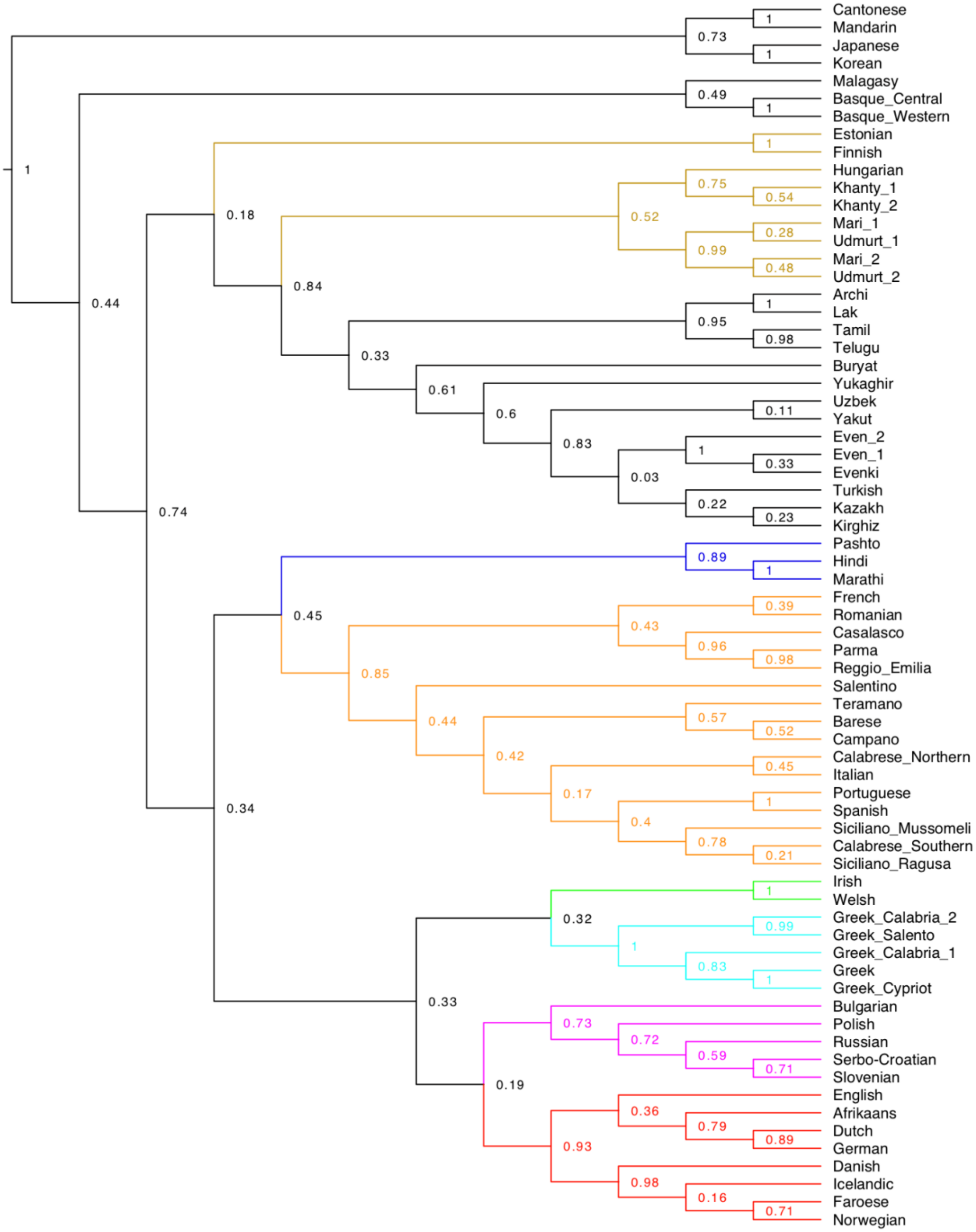

Supplementary Figure 8. Unconstrained BEAST tree.

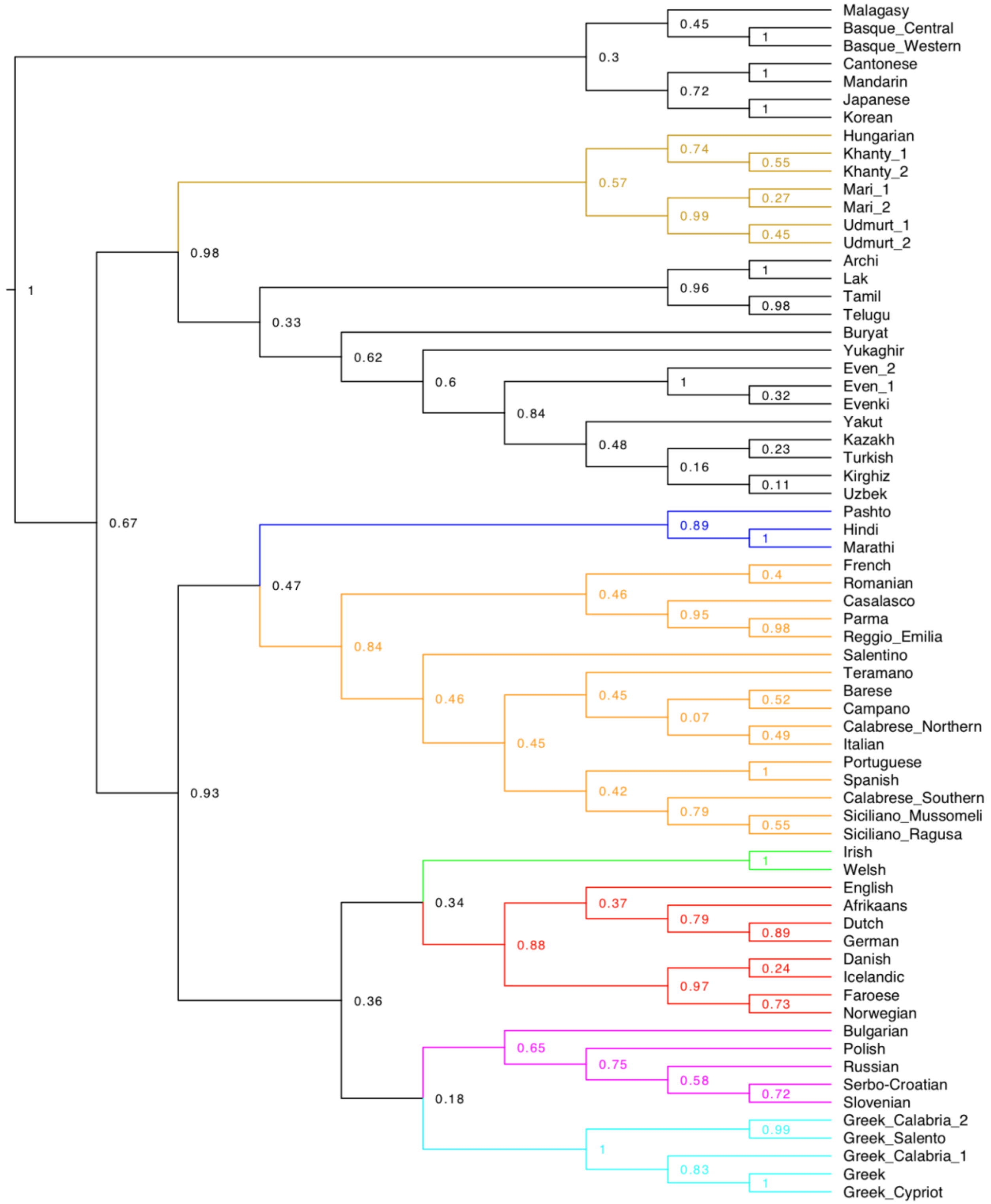

Supplementary Figure 9. BEAST tree without Finnish and Estonian.

10. Network analysis - NeighborNet

For the network analysis, we used the software SplitsTree (Huson and Bryant 2006) and the algorithm NeighborNet. The network (**Supplementary Figure 10**) identifies all the major aggregations already identified in the other experiments. The two graphs containing the Δ-scores (**Supplementary Figure 11**) and the Q-residuals (**Supplementary Figure 12**) have been produced using matplotlib in Python3. **Supplementary Table 5** lists the ten highest Δ-scores and Q-residuals for our dataset.

**Δ-scores**		**Q-residuals**	
Mandarin	0.387	Mandarin	0.125
Cantonese	0.387	Cantonese	0.125
Korean	0.371	Japanese	0.107
Japanese	0.369	Korean	0.098
Pashto	0.367	Hungarian	0.097
Basque_Central	0.365	Lak	0.092
Tamil	0.350	Archi	0.092
Basque_Western	0.349	Basque_Central	0.089
Hungarian	0.336	Basque_Western	0.085
Malagasy	0.336	Tamil	0.081

Supplementary Table 5. The ten highest Δ-scores and Q-residuals for our dataset.

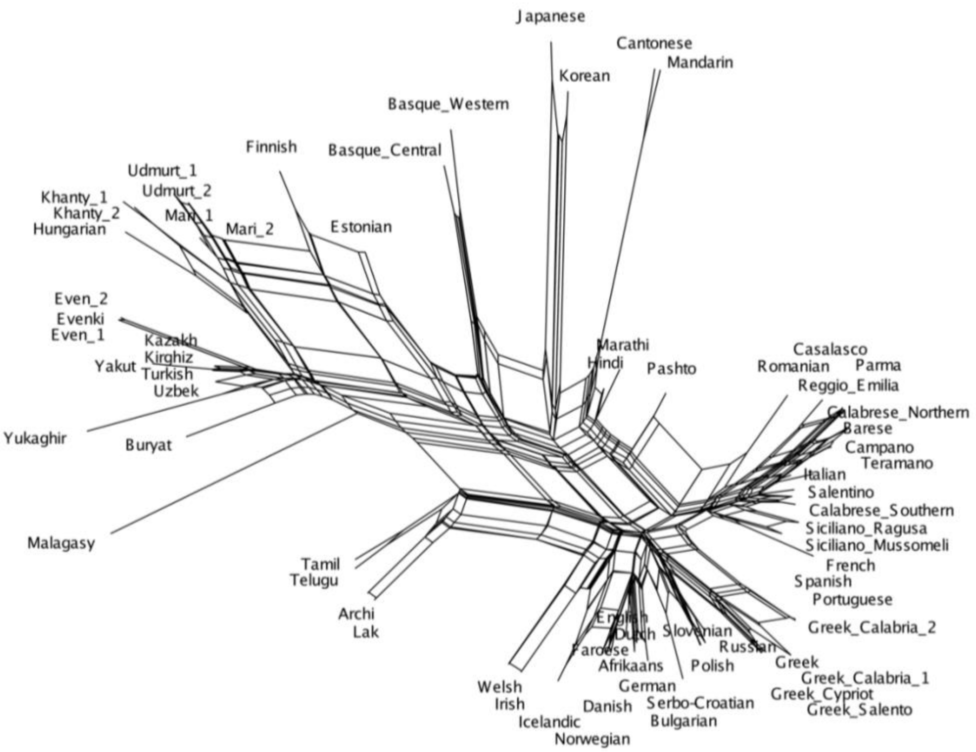

Supplementary Figure 10. NeighborNet network obtained using SplitsTree on the syntactic dataset.

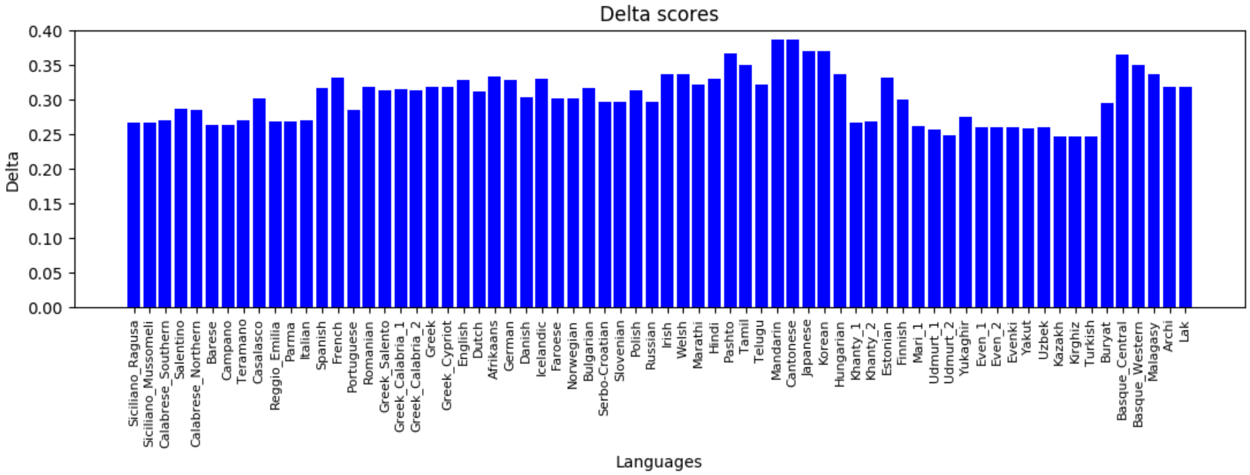

Supplementary Figure 11. Δ-scores derived from the network in **Supplementary Figure 10**.

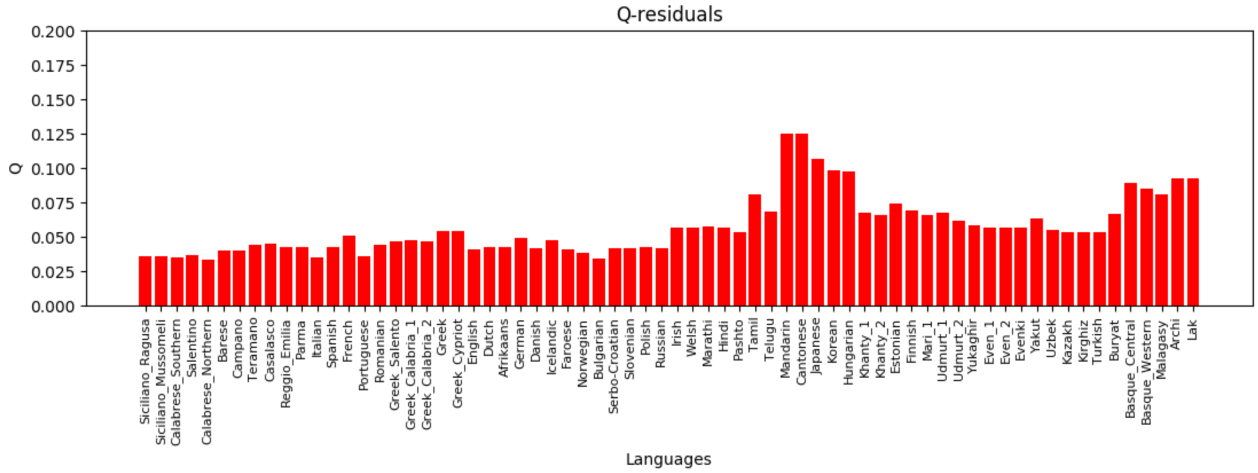

Supplementary Figure 12. Q-residuals derived from the network in **Supplementary Figure 10**.

11. Phonemic data - the Ruhlen Database

The Tracer analysis for the tree generated from Ruhlen's dataset is summarized in **Supplementary Figure 13.** The best model that we determined is a Gamma Site Model with Substitution Rate = 1, a Mutation Death Model with death p = 0.1, a Relaxed Clock (Logarithmic) with clock rate = 1 with clock rate = 1, and a uniform Yule model for the birth rate. The Monte Carlo Markov Chain produced 10,000,000 trees, 25% of which were used for the burn-in and discarded for the purpose of the calculation of the consensus tree. The tree is a consensus tree of 7,500 different trees sampled through the 7,500,000 trees (with a sample stored every 1000 generated trees) produced by Monte Carlo sampling.

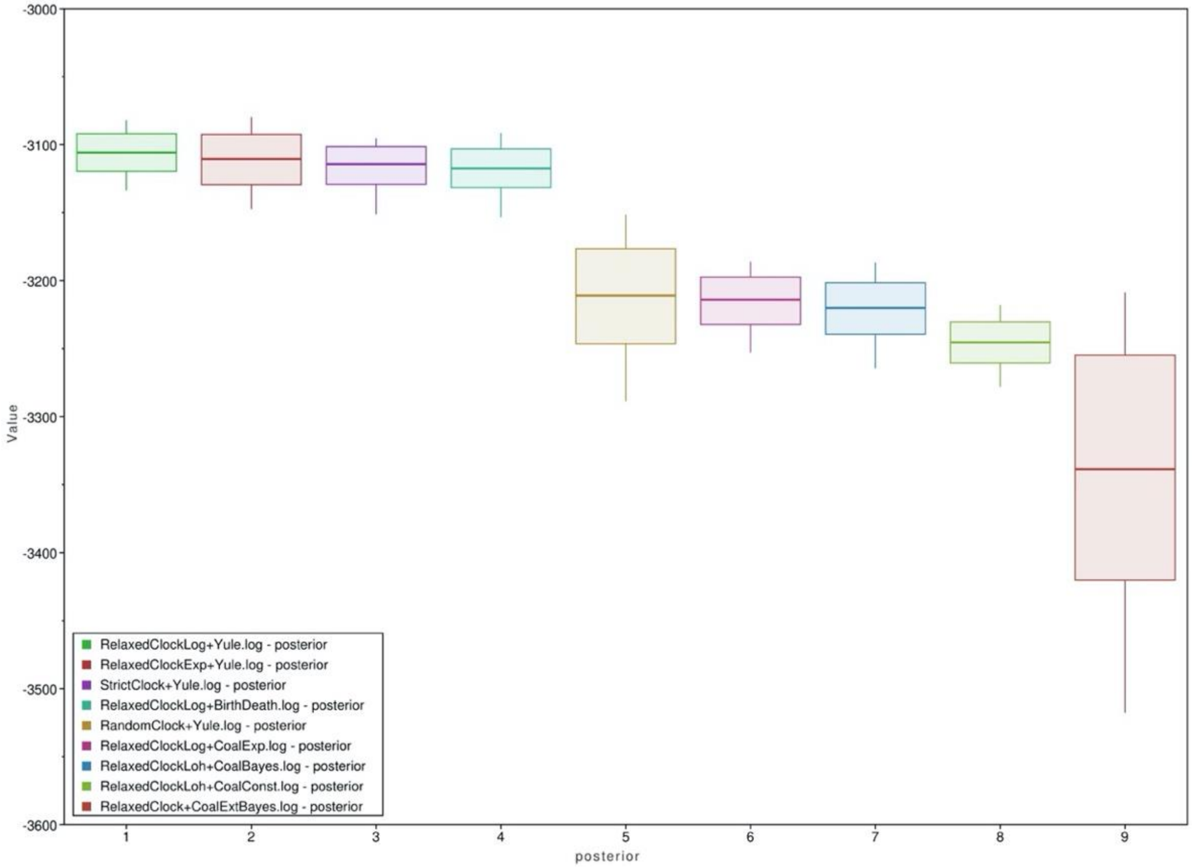

Supplementary Figure 13. Tracer analysis for different BEAST models used to generate a tree from the subset of the Ruhlen dataset overlapping with our languages.

12. Ultralocality

The Network of **Supplementary Figure 14** has been generated from the Romance languages of the sample. Here, the languages of Italy are separated from the rest of Romance, and their internal classification is largely the expected one: the Lausberg dialect (Calabrese_Northern) is an isolate bridging the other Upper southern dialects (Campano, Barese and Teramano) and Italian; the Northern Gallo-Italic group (Teggio_Emilia, Parma and Casalasco) is singled out; the Extreme southern dialects (Siciliano and Calabrese_Southern) are together, with Salentino as the outlier; the position of the Extreme southern group suggests some relation with Ibero-Romance.

In the Heatmap in **Supplementary Figure 15,** white and blue cells mark distances ranging from 0 to 0.142, yellow and red cells mark distances ranging from 0.143 to 0.286.

Instructions to visualize the heatmap:

1. Go to the following page: https://software.broadinstitute.org/morpheus/
2. Upload to the page the file *jaccard_distances.txt* from the GitHub repository (link: https://github.com/AndreaCeolin/FormalSyntax/blob/master/Romance/jaccard_distances_romance.txt), and click the "OK" button to visualize the heatmap.
3. In the "Tools" menu, select the option "Hierarchical clustering", and then the following:
    a. Metric > Matrix values (from a precomputed distance matrix)
    b. Linkage method > average

   c. Cluster > Rows and columns

   Click the “OK” button.

4. To visualize the same color distribution as Fig.1, follow the instructions below:
   a. In the “View” menu, select “Options”
   b. In the “Color Scheme” window:
      i. Uncheck the “Relative color scheme” choice
      ii. “Maximum” > 0.286
      iii. “Add color stop”
      iv. “Selected color” > yellow
      v. “Selected value” > 0.143

Supplementary Table 6 lists the maximal clusters of cells in **Supplementary Figure 15** which do not contain any yellow/red cell. The symbol δ refers to the Jaccard distance between two languages, the symbol μ to the average distance among the languages belonging to a given aggregation/cluster, obtained as the mean of all the pairwise distances between the languages of that aggregation.

**Maximal cluster**	**δ (range)**	**μ/δ**
1. Extreme southern dialects of Italy	From 0 to 0.13	0.06
2. Upper southern dialects of Italy and Italian	From 0 to 0.12	0.07
3. Northern Gallo-Italic dialects	From 0 to 0.08	0.05
4. Ibero-Romance		0.04

Supplementary Table 6. Clusters suggested by the distribution of the distances in the Heatmap in **Supplementary Figure 15**.

The white/blue cells outside of the clusters in **Supplementary Table 6** correspond to the pairs listed in **Supplementary Table 7**.

**Cluster - Language**	**Closer Cluster - Language (δ)**
1 - Siciliano_Ragusa	2 - Italian (0.08), Calabrese_Northern (0.12), Barese (0.12), Campano (0.12)
	4 - Portuguese (0.12)
1 - Siciliano_Mussomeli	2 - Italian (0.08), Calabrese_Northern (0.12), Barese (0.12), Campano (0.12)
	4 - Portuguese (0.12)
1 - Calabrese_Southern	2 - Italian (0.08)
	4 - Portuguese (0.08)
1 - Salentino	2 - Barese (0.13), Campano (0.13), Calabrese_Northern (0.13), Italian (0.13)
2 - Barese	1 - Siciliano_Ragusa (0.12), Siciliano_Mussomeli (0.12), Salentino (0.13)
	3 - Reggio_Emilia (0.13), Parma (0.13)
2 - Campano	1 - Siciliano_Ragusa (0.12), Siciliano_Mussomeli (0.12), Salentino (0.13)
	3 - Reggio_Emilia (0.13), Parma (0.13)
2 - Teramano	3 - Reggio_Emilia (0.08), Parma (0.08)
2 - Calabrese_Northern	1 - Siciliano_Ragusa (0.12), Siciliano_Mussomeli (0.12), Salentino (0.13)
2 - Italian	1 - Siciliano_Ragusa (0.08), Siciliano_Mussomeli (0.08), Calabrese_Southern (0.08), Salentino (0.13)
	3 - Reggio_Emilia (0.12), Parma (0.12)
	4 - Spanish (0.14)
	Romanian (0.14)
3 - Reggio_Emilia	2 - Barese (0.13), Campano (0.13), Teramano (0.08), Italian (0.12)

3 - Parma	2 - Barese (0.13), Campano (0.13), Teramano (0.08), Italian (0.12)
4 - Portuguese	1 - Siciliano_Ragusa (0.12), Siciliano_Mussomeli (0.12), Calabrese_Southern (0.12)
	2 - Italian (0.11)
4 - Spanish	2 - Italian (0.14)
(isolate) Romanian	2 - Italian (0.14)

Supplementary Table 7. White/blue cells in **Supplementary Figure 15** outside of the clusters listed in **Supplementary Table 6**.

In the PCoA in **Supplementary Figure 16,** the vertical axis (43% of the variance) separates Ibero-Romance and the dialects of central/southern Italy from the rest of Romance, with the exception of Teramano; Barese and Campano fall precisely on the vertical axis. The horizontal axis (36% of the variance) separates the dialects of Italy from the other Romance languages, with two exceptions: Calabrese_Southern (that appears right below the axis), and Casalasco (the Northern Gallo-Italic dialect closest to French).

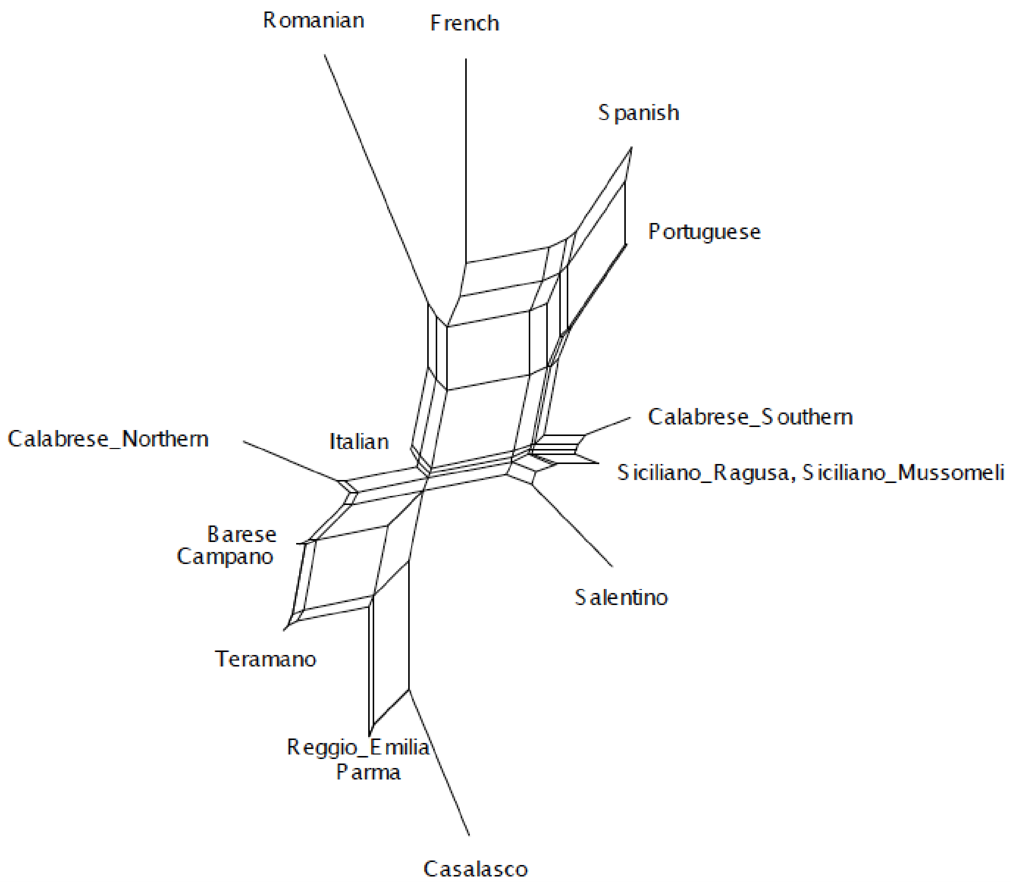

Supplementary Figure 14. Network of the Romance languages.

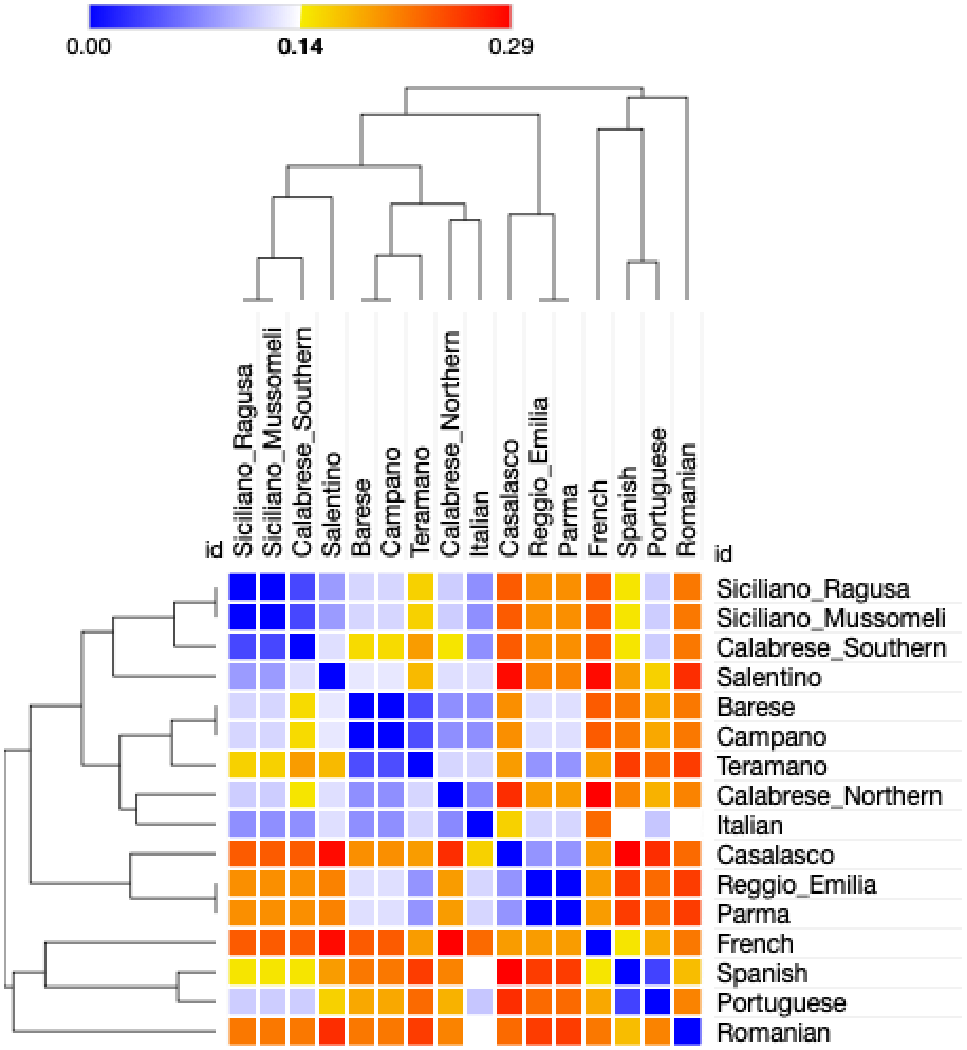

Supplementary Figure 15. Heatmap of the Romance languages.

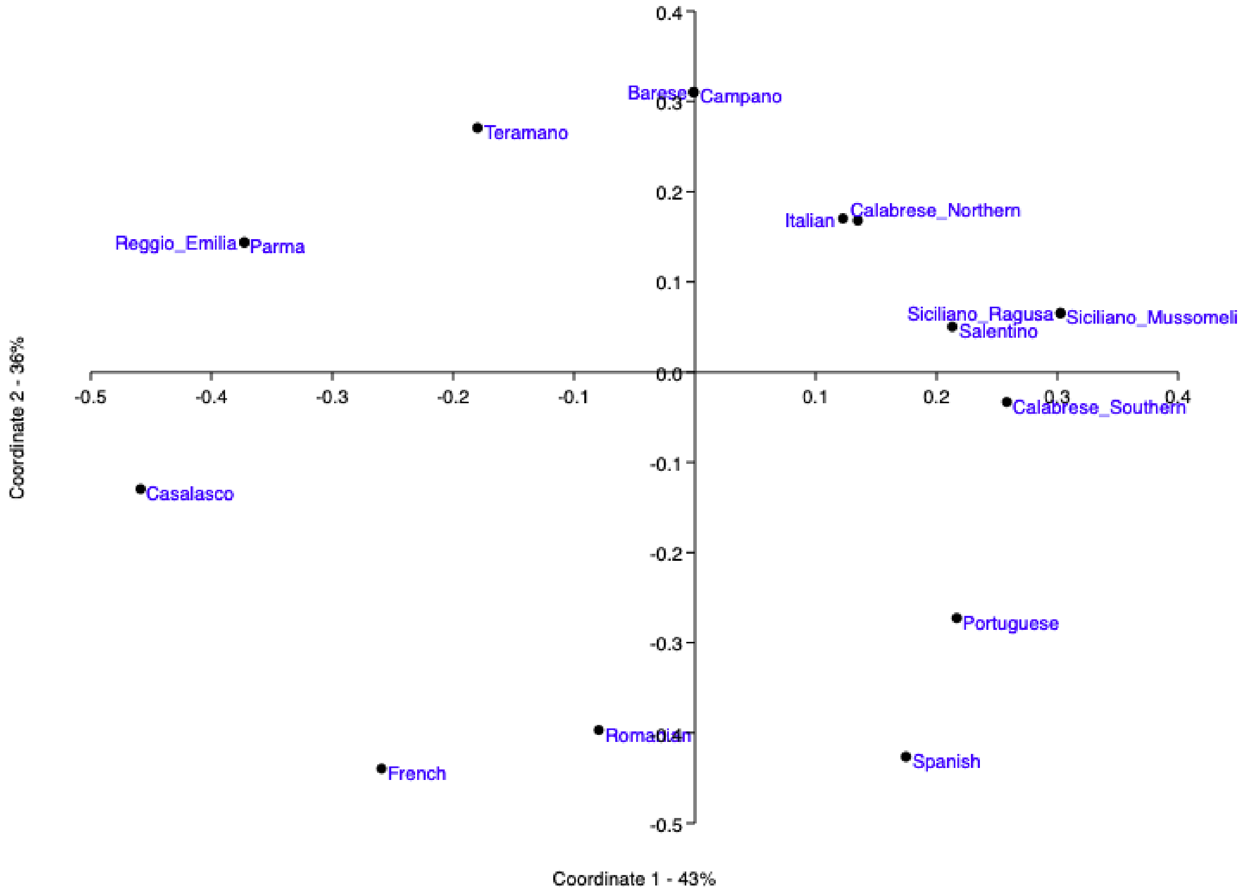

Supplementary Figure 16. PCoA of the Romance languages.
